# 16-nor Limonoids from *Harrisonia perforata* as promising selective 11*β*-HSD1 inhibitors

**DOI:** 10.1038/srep36927

**Published:** 2016-11-11

**Authors:** Xiao-Hui Yan, Ping Yi, Pei Cao, Shi-Ying Yang, Xin Fang, Yu Zhang, Bin Wu, Ying Leng, Ying-Tong Di, Yang Lv, Xiao-Jiang Hao

**Affiliations:** 1 State Key Laboratory of Phytochemistry and Plant Resources in West China, Kunming Institute of Botany, Chinese Academy of Sciences, Kunming 650201, P. R. China; 2 College of Forestry, Southwest Forestry University/Key Laboratory of Forest Disaster Warning and Control of Yunnan Province, Kunming 650224, P. R. China; 3 Key Laboratory of Chemistry for Natural Products of Guizhou Province and Chinese Academy of Sciences, Guiyang 550002, P. R. China; 4 Beijing Key Laboratory of Polymorphic Drugs, Institute of Materia Medica, Chinese Academy of Medical Sciences & Peking Union Medical College, Beijing 100050, P. R. China; 5 Shanghai Institute of Materia Medica, Chinese Academy of Sciences, Shanghai 200031, P. R. China

## Abstract

Two new 16-nor limonoids, harperspinoids A and B (**1** and **2**), with a unique 7/5/5/6/5 ring system, have been isolated from the plant *Harrisonia perforate* together with a known one, Harperforin G (**3**). Their structures were elucidated by NMR spectroscopy, X-ray diffraction analysis and computational modelling. Compound 1 exists as polymorphic crystals. Conformations of **1** in solution were further discussed based on the computational results. These compounds exhibited notable inhibitory activity against the 11*β*-HSD1 enzyme. Compound **3** had potencies for the inhibition of human 11*β*-HSD1 with high selectivity against 11*β*-HSD2 (IC_50_ 0.58 *μ*M, SI > 174). Molecular docking and quantitative structure-activity relationship studies revealed a mixed regulatory mechanism.

11*β*-Hydroxysteroid dehydrogenase type 1 (11*β*-HSD1) is the enzyme primarily responsible for the regulation of intracellular cortisol levels^1^,^2^. Inhibition of 11*β*-HSD1 is an attractive therapeutic approach for the treatment of obesity and other elements of metabolic syndrome, such as type 2 diabetes mellitus^3^. Up to now, a number of potent and selective 11*β*-HSD1 inhibitors have been reported, some of which are progressing in different phases of clinical trials^4^. However, most of the candidates are synthetic chemicals, and natural compounds or their derivatives with highly promising selectivity are still scarce^5^.

Limonoids with diverse structures and significant bioactivities have become a hot topic in the field of natural products and synthetic chemistry^6^. Their occurrence in the plant kingdom is confined mainly within the Meliaceae and Rutaceae families and less frequently within the Cneoraceae, Ptaeroxylaceae, and Harrisonia genera of Simaroubaceae^6^,^7^. *Harrisonia perforata* is the only species of this genus grown in China, and its root and leaves have been applied in Chinese folk medicine for the treatment of wound healing and malaria^8^. Previous investigations of the chemical constituents of this plant have revealed an array of structurally diverse chromones, quassinoids, polyketides, and highly rearranged limonoids^9^,^10^,^11^,^12^,^13^. Recently, unprecedented quassinoids and limonoid derivatives with notable biological properties have been discovered and evaluated by our group^14^,^15^,^16^. As part of our continuous effort to search for bioactive natural products^17^,^18^,^19^,^20^, two new 16-nor limonoids, harperspinoids A and B (**1** and **2**), with a unique 7/5/5/6/5 ring system, as well as a known one, Harperforin G (**3**), were isolated from the aerial parts of the title plant (Fig. 1). Herein, the isolation, structural elucidation, and inhibitory effects of **1**–**3** on 11*β-*HSD1 are described.

## Results and Discussion

The air-dried plant material powder (25.0 kg) was extracted with MeOH three times, and the combined extracts were concentrated, followed by suspension in water. The water layer was then extracted with petroleum ether and EtOAc. Isolation of the EtOAc extracts (560 g) yielded compounds **1** (26 mg), **2** (5.9 mg), and **3** (37 mg).

Harperspinoid A (**1**) was obtained as colourless crystals with the specific rotation [*α*]_D_^16^ +27.3. It possessed a molecular formula C_25_H_28_O_7_ with 12 degrees of unsaturation, as deduced from HRESIMS (*m/z* 463.1744 [M + Na]^+^; calcd 463.1732). The IR absorption bands showed the existence of carbonyl groups (1758 and 1704 cm^–1^). The NMR data including DEPT and HSQC spectra revealed the presence of four methyls, three methylenes, nine methines (five olefinic ones), and nine quaternary carbons (three olefinic ones and two carbonyls) (Table 1). Further analysis of 1D NMR demonstrated the presence of four tertiary methyls (*δ*_H_ 0.91, H_3_-18; 1.46, H_3_-28; 1.52, H_3_-19 and H_3_-29; *δ*_C_ 21.2, C-18; 28.1, C-28 and C-29; 31.2, C-19), one ketal group (*δ*_C_ 111.2, C-7), one disubstituted *α*,*β*-unsaturated ester moiety (*δ*_H_ 5.86, H-2; 6.21, H-1; *δ*_C_ 119.9, C-2; 146.4, C-1; 165.6, C-3), one tetrasubstituted *α, β*-unsaturated ester moiety (*δ*_C_ 125.3, C-14; 146.5, C-8; 168.1, C-15), and one *β*-furan group (*δ*_H_ 6.28, H-22; 7.42, H-23; 7.43, H-21; *δ*_C_ 108.1, C-22; 119.8, C-20; 139.7, C-21; 143.6, C-23), accounting for seven degrees of unsaturation. The remaining five degrees of unsaturation suggested that compound **1** is pentacyclic. All the information mentioned above indicated that **1** should be a 16-nor limonoid derivative^14^.

By extensive interpretation of ^1^H-^1^H COSY and HMBC spectra, the planar structure of harperspinoid A was established (Fig. 2). In HMBC spectrum, the correlations of H_3_-18/C-12, C-13, C-14,and C-17; H-9/C-8, and C-14; and H-17/C-13, C-14, C-15, C-20, C-21, and C-22 indicated the presence of an octhydroisobenzo-furan moiety (rings D and E) with a double bond between C-8 and C-14, a methyl at C-18, a ketone carbonyl at C-15, and a *β*-furan moiety at C-17 (in purple)^14^. The cross peaks of H-2/C-3; H-19/C-1, C-5, C-10; H_3_-28(29)/C-4, and C-5, and H-5/C-3 (*J*^4^) in HMBC spectrum defined the formation of ring A (in green). In addition, the COSY connectivity (Fig. 2) between *δ*_H_ 2.92 (H-5) and *δ*_H_ 2.17/2.34 (H_2_-6) indicated a C5–C6 spin system. The HMBC correlations from H_2_-6 and H_2_-30 to the ketal carbon (*δ*_C_ 111.2, C-7), together with the HMBC connectivity of H_2_-6 with C-10 and C-30, and H_2_-30 with C-8 and C-9, along with the downfield shifted C-9 (*δ*_C_ 76.8, oxygenated), indicated the rearranged B ring was a spirocyclic moiety. This spirocyclic moiety contained two oxygenated five-member rings, B1 and B2, with two oxygenated carbons C-4 and C-9 attached to the C-7 ketal (in red). Thus, the planar structure of **1** was established, which is the first example of a 16-nor limonoid with a 7/5/5/6/5 skeleton.

The relative configuration assigned for **1** was deduced by the analysis of ROESY data. Examination of a Dreiding molecular model of **1**, suggested that **1** adopts a conformation in which both of the five-member rings, B1 and B2, is orthogonal to each other. ROESY correlations of H-30*β*/H-9/H-12*β*/H-17 indicated that H-30*β*, H-9, and H-17 are all on the same face of the octhydroisobenzo-furan moiety, thus, orienting them toward oxygen atom of ring B1. ROESY interactions of H-30*α* with H_2_-6, H-6*α* with H-5, and H-5 with Me-19 placed the corresponding substituents together on the opposite face of the octhydroisobenzo-furan ring, and fixing the relative configuration at C-7 as shown.

Needle and prism shaped crystals of **1** were obtained simultaneously from MeOH/H_2_O (9:1) via slow evaporation. The single-crystal X-ray diffraction analysis of each sample showed that **1** exists in two crystalline forms, termed **HA** and **HB**, respectively (deposition no. CDDD 999635 and 999636, https://www.ccdc.cam.ac.uk/services/structure_deposit/) (Fig. 3). Form **HA** crystallizes in the monoclinic *P*2_1_ space group. Rings A and D adopt a half-chair conformation; rings B, C, and E take the envelope conformation; and the *β*-furan ring is almost planar and adopts a *cis*-conformation with a dihedral angle of 24.5°. In contrast to **HA**, form **HB** crystallizes in the triclinic, *P*2_1_2_1_2_1_ space group. Ring A takes a boat conformation, and the *β*-furan ring is arranged in a *trans*-conformation with a dihedral angle of −125.8°. To the best of our knowledge, this is the first case of limonoids with polymorphism. Moreover, the final refinement of **HA** on the Cu Kα data resulted in a Flack parameter of 0.2(2), which gave an unambiguous assignment of the absolute configuration of **1** as (5 S, 7 S, 9 R, 10 R, 13 R and 17 S)^21^.

As an extensive exploration of all the conformations of **1** in solution, a computational modelling study was conducted using Gaussian-03 program at the B3LYP/6–31 G* level (Gaussian-03, revision D.01, Gaussian Inc., Pittsburgh). The calculations showed four low-energy conformations of **1**, which were roughly distinguished as boat, trans (**BT**), half-chair, cis (**HCC**), half-chair, trans (**HCT**), and boat, cis (**BC**) according to the conformational difference of ring A and the orientation of the *β*-furan ring (Fig. 4). Then, we compared bond lengths, bond angles, and dihedral angels of **HCC** and **BC** with those of the crystal structure in the forms **HA** and **HB**, and their RMS value are calculated to be 0.4985. All these data indicate that calculated **HCC** and **BC** are in good agreement with **HA** and **HB**, respectively. Moreover, the calculation also showed two transition states (**TS1** and **TS2**) (Fig. 5), which corresponded to the conformational conversion of ring A and the rotation of the *β*-furan ring; their free energies against the most stable conformer **HCC** were 17.7 and 4.9 kcal/mol, respectively. Therefore, the occurrence of rapid interconversion of the four conformers of **1** is a logical process in solution at room temperature^22^,^23^,^24^ (for details, see the supporting information, SI). Furthermore, the stability of the two polymorphs was calculated using a molecular mechanics method performed using SYBYL 8.1. Molecular energy of the two crystal polymorphs of **1** is 260.1 kcal/mol (**HA**) and 258.1 kcal/mol (**HB**), respectively. The small energy difference between them indicated that the two conformers of **1** could simultaneously assemble into different crystal at room temperature^23^.

**Figure 1:**
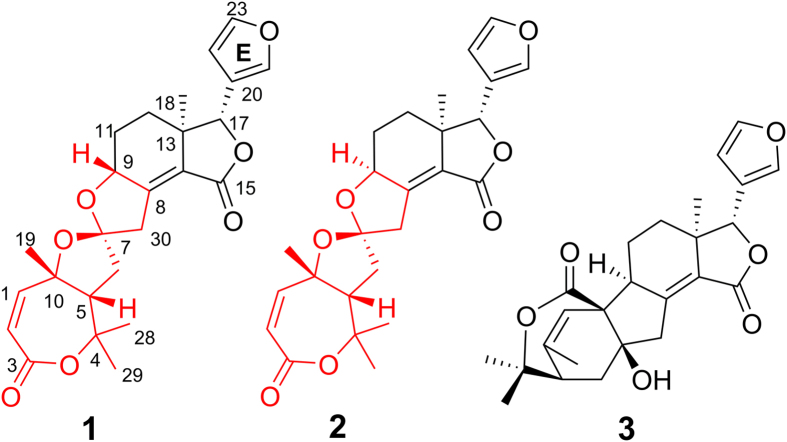
Chemical structures of 1–3.

**Figure 2:**
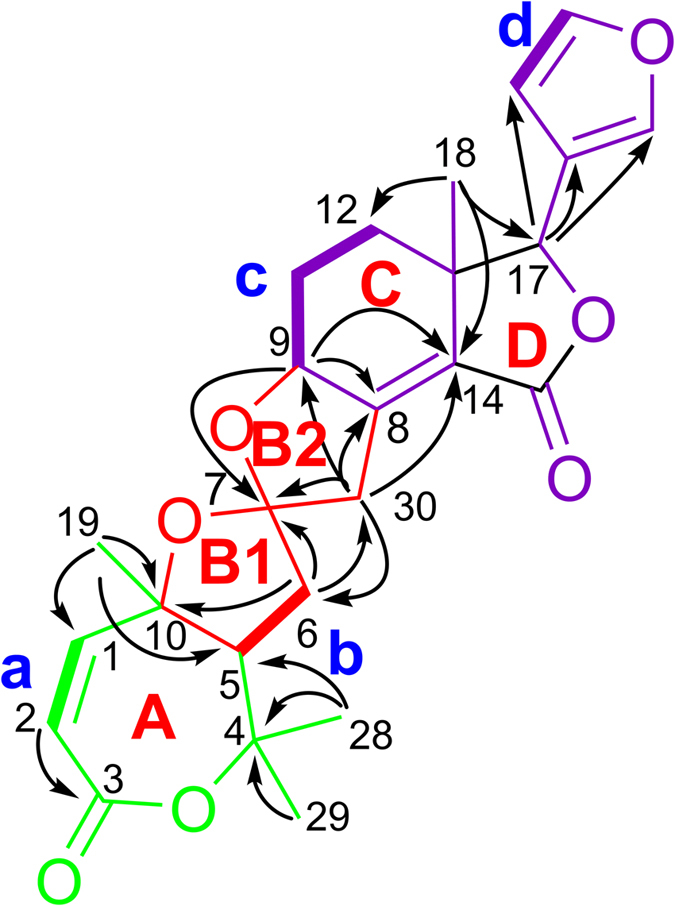
Key ^1^H–^1^H COSY (bold), HMBC (arrow) correlations of compound 1.

**Figure 3:**
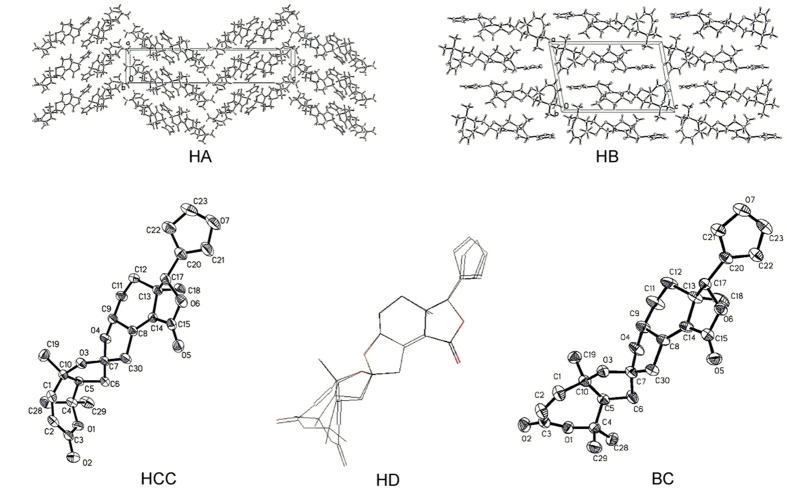
Single-crystal X-ray structure of 1: two crystal packings (HA and HB), two conformational polymorphs (HCC and BC), and an overlay (HD) of the two conformation of 1.

**Figure 4:**
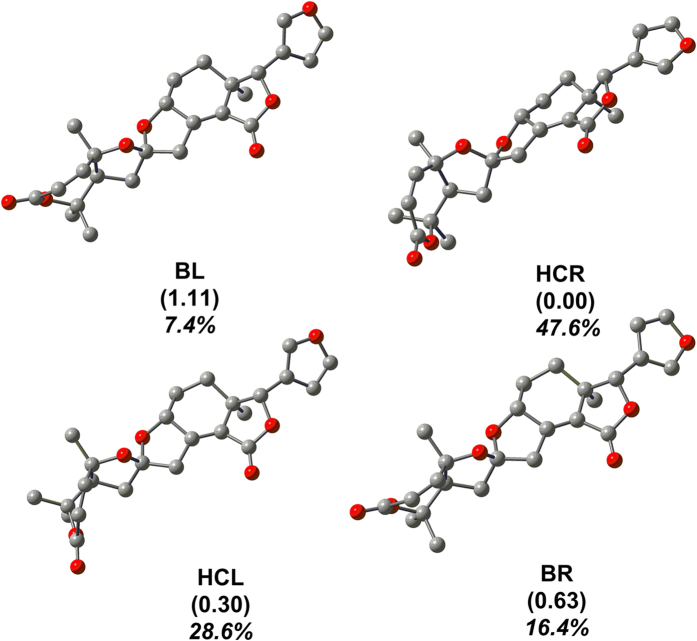
Optimized geometries of the 4 conformations (BT, HCC, HCT and BC) of 1 at the B3LYP/6-31 G* level in the gas phase. Free energies in kcal/mol relative to the most stable conformation, HCR, are given in parentheses. Populations of the four conformations (in italic) are also given.

**Figure 5:**
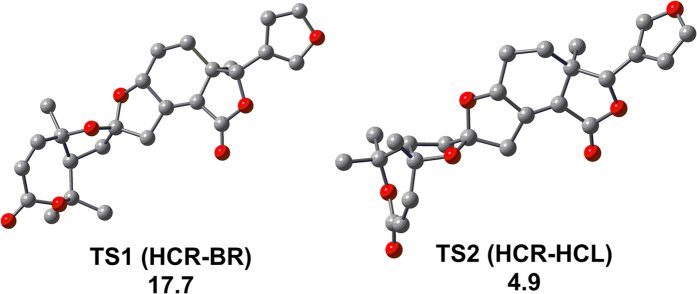
DFT-calculated two connecting transition states (TS1 and TS2). Free energies in kcal/mol relative to the most stable conformer of 1, HCR, are also given.

**Figure 6:**
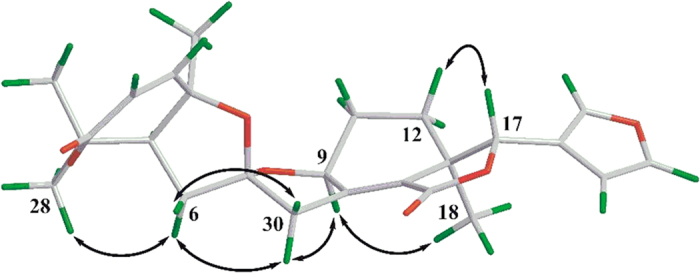
Key ROESY (arrow) correlations of 2.

Harperspinoid B (**2**) had the molecular formula C_25_H_28_O_7_ based on HRESIMS, which is the same as that of **1**. The NMR features of **2** (Table 1) closely resembled those of **1** except for the resonances near C-9. The data from the ^1^H-^1^H COSY, HSQC and HMBC spectra indicated that compound **2** shared the same planar structure as that of **1**. The ROE correlation of Me-18 (*δ*_H_ 0.92)/H-9 (*δ*_H_ 4.39) suggested that H-9 is *α*-orientated. In addition, the relative configuration of the remaining chiral centres of **2** would be analogous to those of **1** based on ^13^C NMR shifts and NOE data (Fig. 6). On the basis of biogenetic considerations, the absolute configuration of **2** is tentatively assigned as 5 S, 7 S, 9 S, 10 R, 13 R and 17 S. Thus, the structure of **2** was eventually established as shown in Fig. 1.

**Figure 7:**
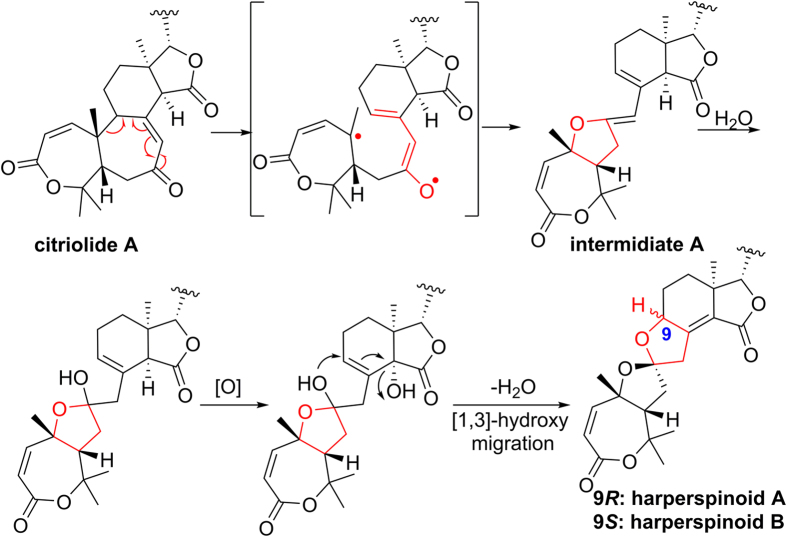
Proposed biogenetic pathway of compounds 1 and 2.

Biogenetically, compounds **1** and **2** might be derived from Citriolide A, which may be converted to the key hemiketal intermediate A via a free radical mechanism. Subsequently, the intermediate A may undergo oxidation, cyclization, and double-bond migration in turn to generate **1** and **2** (Fig. 7).

The inhibitory activity of compounds **1**–**3** on murine and human 11*β*-HSD1 was evaluated using the scintillation proximity assay (SPA)^25^. In intact CHOP cells transfected with murine *HSD11B1*, only compound **1** showed inhibitory effects with an IC_50_ value of 0.60 μM. Moreover, compound **1** were highly selective against murine 11*β*-HSD2 activity since it did not inhibit the enzyme at all at 1 mM (SI >1661). We further used the intact cell transfected with human *HSD11B1* and *HSD11B2* to screen their bioactivities. Compound **3** showed high potency for selective inhibition of human 11*β*-HSD1 (IC_50_ 0.58 *μ*M and SI >174).

**Figure 8:**
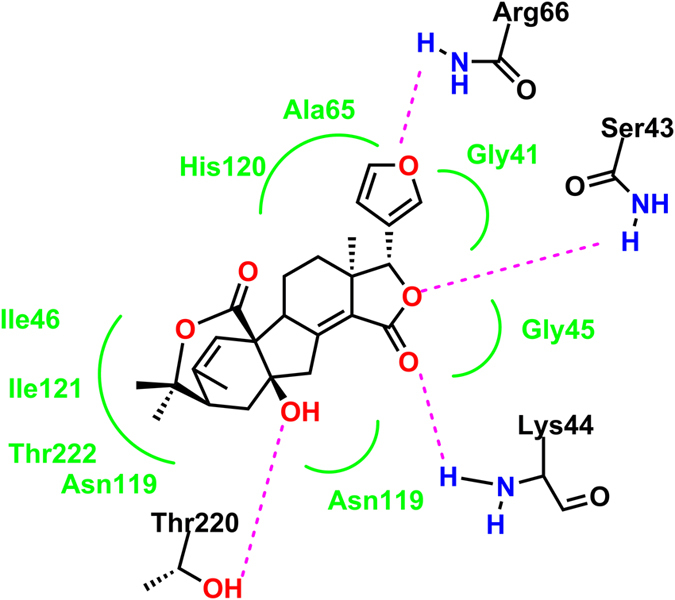
Structural binding environment of compound 3 to the 11*β*-HSD1 enzyme (4K1L for human). Amino acid chains close to the binding sites around ligand 3 are shown.

To better understand the structure-activity relationship of the compounds, a molecular docking simulation was performed using co-crystal structures of the 11*β*-HSD1 enzyme (4K1L for human). Despite the structural variety of the different inhibitors that have previously been investigated, the crystal structures of the NADP(H)-dependent 11*β*-HSD1 proteins are comparatively similar^26^. AutoDock 4 was employed to quantify the parameters that are crucial for high affinity ligand binding. According to the three dimensional images, compounds **1**, **2** and **3** could occupy part of the active sites in cofactor NADP(H) with docking binding energies of −9.63, −9.81 and −10.19 kcal/mol, respectively. The order of the binding energy followed the inhibitory trend for the above three substrates. LigPlot+ was then used for further analysis of the complex between compound **3** and the enzyme. In the docking model, **3** adopted a V shape to fit well into the hydrophobic pocket of the receptor (Fig. 8). In addition to the hydrophobic interaction due to the nature of the polycyclic aliphatic skeleton bearing a furan unit, the hydrogen bonding interactions induced by Arg66, Ser43, Lys44 and Thr220, together with the adjacent *O*-containing functional groups surrounding **3** could significantly enhance the affinity.

In contrast to the general 11*β*-HSD1 inhibitors, which form the key hydrogen bonding interactions through Tyr183 and Ser170 within the active site^26^, compound **3** not only lodges in the usual anchoring position and participates in interactions with unusual catalytic residues but also encroaches partially on the cofactor site. NADP(H) specificity in 11*β*-HSD1 is achieved through the packing interaction with Lys44, a hydrogen bond between its 3′-OH and Ser43, and an electrostatic interaction of the ribose 2′-phosphate with guanidinium N atoms in Arg66; at the same time, there is additional contact to this 2′-phosphate by the backbone amide of Arg66^27^. The competitive interactions of compound **3** with Arg66, Ser43, and Lys44, which comprise crucial residues for electrostatic interaction and H-bond formation involved with NADP(H) specific localizations in 11*β*-HSD1,would obviously attenuate or reduce their corresponding interactions with NADP(H). However, the reimbursements were most likely provided by the emerging interactions within the optimum approach distance between NADP(H) and the invasive ligand **3**. Thus, the unitary binding affinity seems to be retained. In this special manner with synergistic effects, the high inhibitory activity of incorporating compound **3** could be explained more reasonably.

In summary, we have isolated and identified three 16-nor limonoids, including two new ones from the aerial parts of *H. perforata*. Two polymorphic forms of harperspinoid A (**1**) were discovered and unambiguously characterized. Its conformers and their interconversion process in solution were further discussed based on computational modelling. Compound **3**, the most potent one, had an IC_50_ of 0.58 *μ*M in a whole cell assay. As illustrated through the docking simulation of compounds **1**–**3** with 11*β*-HSD1 (4K1L for human), the structural analogues **1**–**3** probably inhibit 11*β*-HSD1 in a mixed manipulating pattern. They might occupy the common locating pocket and compete for the catalytic residues that affect NADP(H) binding while generating compensatory interactions via the invasion of the active sites in this cofactor. The unexpected dual modulation of compounds **1**–**3** on both the substrate and NADP(H) bindings is worthy of further investigation, which might be an interesting objective for future exploration. Our present discovery has demonstrated the versatility and elegance of regulating mechanisms relating to traditional 11*β*-HSD1 accompanied by its cofactor and supplied valuable information for the design of novel alternative inhibitors.

## Methods

### General experimental procedures

Crystal data were measured using a Cu Kα radiation (graphite monochromator). Optical rotations were determined with a Perkin-Elmer 241 polarimeter. IR spectra were recorded on a Bio-Rad FTS-135 spectrometer with a KBr disk. 1D NMR and 2D NMR were recorded on a Bruker AM-400 spectrometer and a Bruker DRX-500 instrument. ESIMS and HRESIMS spectra were measured with a Finnigan MAT 90 instrument and VG Auto Spec-3000 spectrometer, respectively. Semi preparative HPLC was performed on a Merck column (i.d. 100–10 mm; Merck, Darmstadt, Germany). MCI gel (CHP20P, 75–150 *μ*m, Mitsubishi Chemical Industries Ltd.); Sephadex LH-20 (40–70 *μ*m; Amersham Pharmacia Biotech AB, Uppsala, Sweden); Column chromatography was performed on silica gel (90–150 *μ*m; Qingdao Marine Chemical Inc.); TLC plates were precoated with silica gel GF_254_ and HF_254_ (Qingdao Haiyang Chemical Plant, Qingdao, People’s Republic of China). Fractions were monitored by TLC and spots were visualized by heating silica gel plates sprayed with 10% H_2_SO_4_ in EtOH.

### Plant Material

The leaves and branches of *Harrisonia perforata* collected from Hainan province of China in November 2008, and authenticated by Dr. Hao-Fu Dai of Chinese Academy of Tropical Agricultural sciences. A voucher specimen (accession number KIB-20081102) has been deposited in Kunming institute of botany.

### Extraction and Isolation

The air-dried powder of the leaves and branches of *Harrisonia perforata* (25.0 kg) was extracted with MeOH three times, followed by combination, concentration, and suspension in water. It was subsequently partitioned successively with PE (petroleum ether), EtOAc, and nBuOH. The EtOAc part (560 g) was chromatographed on a silica gel column eluted with PE/acetone (from 1:0 to 0:1) to give 6 fractions (A1–A6). A3 (PE/acetone5:1–3:1, 17 g) was fractionated via an MCI gel column eluted with gradient 80% MeOH/H_2_O and further separated by Sephadex LH–20 (MeOH) recrystallization in methanol to afford **3** 37 mg. A4 (PE/acetone 3:1, 150 g) was fractionated via an MCI gel column eluted with gradient MeOH/H_2_O from 5:5 to 9:1 to obtain five fractions (B1–B5). Fraction B2 (28 g) was subjected to Sephadex LH–20 (MeOH) to afford (C1-C4) four elutes. Fraction C2 (6.1 g) was subjected to CC with C18 reversed-phase silica gel (MeOH/H_2_O = 30:70–100:0) followed by extensive CC over columns of LH-20 and silica gel yield a mixture of **1** and **2** (40 mg),which was further separated by semi preparative HPLC (MeOH/H_2_O, 55:35, 3 ml/min) to yield **1** (26 mg) and **2** (5.9 mg).

### Calculation Methodology

All calculations were performed using the Gaussian 03 program package. Geometries were fully optimized with the density functional theory methods of B3LYP at the 6–31 G* level. Only one negative eigenvalue and one imaginary frequency were obtained for TSs in computations. Intrinsic reaction coordinates (IRC) were also calculated to authenticate the transition state. The free energy magnitudes were used throughout the theoretical studies.

### 11*β*-HSD Enzyme Activity Assay

The inhibitory activities of the compounds on human or mouse 11*β*-HSD1 and 11*β*-HSD2 were determined using the scintillation proximity assay (SPA). The full-length cDNAs of human or murine11*β*-HSD1 and 11*β*-HSD2 were isolated from the cDNA libraries provided by NIH Mammalian Gene Collection. The cDNAs were cloned into pcDNA3 expression vectors. HEK-293 cells were transfected with the pcDNA3-derived expression plasmid and selected by cultivation in the presence of 700 *μ*g/ml of G418. The microsomal fraction overexpressing 11*β*-HSD1 or 11*β*-HSD2 was prepared from the HEK-293 cells, which were stably transfected with 11*β*-HSD1 or 11*β*-HSD2. The fraction was then used as the enzyme source for SPA. Microsomes containing human or mouse 11*β*-HSD1 were incubated with NADPH and [^3^H] cortisone. The product, [^3^H] cortisol, was specifically captured by a monoclonal antibody coupled to protein A-coated SPA beads. The 11*β*-HSD2 screening was performed by incubating 11*β*-HSD2 microsomes with [^3^H] cortisol and NAD + and monitoring substrate disappearance. All tests were done twice with glycyrrhizinic acid as a positive control. IC_50_ (X + SD, n = 2) values were calculated by using Prism Version 4 (GraphPad Software, SanDiego, CA).

### 11*β*-HSD Enzyme Docking Assay

We searched for a possible binding sites for the compounds (**1-3**) on 11β-HSD enzyme using the AutoDock4 docking program and the structure of 11*β*-HSD enzyme (Protein Data Bank 4K26 and 4K1L) as the receptor molecules. The docking studies of these compounds with human (4K1L) and murine (4K26)11*ß*-HSD1 enzymes have been performed respectively. Further analyses using LigPlot+ program revealed the Interactions of the ligand and the enzymes.

### Physic-chemical Characters of the New Compounds

Harperspinoid A (**1**): colorless crystal (MeOH); mp 195–197 °C (**HA**, prism)/mp 176–177 °C (**HB**, needle); [α]^16^_D_ +27.3 (*c* 0.11, MeOH); IR (KBr) *v*_max_ 2946, 1758, 1704, 1643, 1291, 1123 and 930 cm^–1^; positive-ion ESIMS *m/z* 463.3 [M + Na]^+^; HRESIMS *m/z* 463.1744 [M + Na]^+^, calcd 463.1732; ^1^H and ^13^C NMR data, see Table 1.

Harperspinoid B (**2**): white powder; [α]^27^_D_ = −62.8 (*c* 0.36, MeOH); IR (KBr) *v*_max_ 2933, 1760, 1704, 1641, 1449, 1284, 1164 and 995 cm^–1^; positive-ion ESIMS *m/z* 463.3 [M + Na]^+^; HRESIMS *m/z* 463.1721 [M + Na]^+^, calcd 463.1732; ^1^H and ^13^C NMR data, see Table 1.

## Additional Information

**How to cite this article**: Yan, X.-H. *et al*. 16-nor Limonoids from *Harrisonia perforata* as promising selective 11*β*-HSD1 inhibitors. *Sci. Rep.* **6**, 36927; doi: 10.1038/srep36927 (2016).

**Publisher’s note:** Springer Nature remains neutral with regard to jurisdictional claims in published maps and institutional affiliations.

## Supplementary Material

Supplementary Information

## Figures and Tables

**Table 1:** NMR Data for compounds 1 and 2 in CDCl_3_ (*δ* in ppm, *J* in Hz).

No.	1		2	
	*δ*_H_	*δ*_C_	*δ*_H_	*δ*_C_
1	6.21 (d, 12.5)	146.4 (d)	6.16 (d, 12.5)	146.4 (d)
2	5.86 (d, 12.5)	119.9 (d)	5.86 (d, 12.5)	119.9 (d)
3	—	165.6 (s)	—	165.7 (s)
4	—	79.9 (s)	—	79.8 (s)
5	2.92^a^	56.6 (d)	2.91^a^	56.8 (d)
6	α 2.17^a^	39.7 (t)	α 2.38 (m)	38.5 (t)
	β 2.34 (m)		β 2.24 (m)	
7	—	112.2 (s)	—	111.4 (s)
8	—	146.5 (s)	—	152.3 (s)
9	4.52 (m)	76.8 (d)	4.39 (m)	77.3 (d)
10	—	83.2 (s)	—	83.2 (s)
11	α 1.72 (m)	23.7 (t)	α 2.18 (m)	31.25 (t)
	β 2.16^a^		β 1.53 (m)	
12	α 1.87 (m)	30.4 (t)	α 1.89 (m)	28.5 (t)
	β 1.75 (m)		β 1.43 (m)	
13	—	43.4 (s)	—	43.7 (s)
14	—	125.3 (s)	—	127.4 (s)
15	—	168.1 (s)	—	168.5 (s)
17	4.96 (br s)	83.5 (d)	5.05 (s)	81.7 (d)
18	0.91 (s)	21.2 (q)	0.92 (s)	23.6 (q)
19	1.52 (s)	31.2 (q)	1.51 (s)	31.2 (q)
20	—	119.8 (s)	—	119.6 (s)
21	7.43 (br s)	139.7 (d)	7.49 (s)	140.2 (d)
22	6.28 (br s)	108.1 (d)	6.39 (s)	108.7 (d)
23	7.42 (br s)	143.6 (d)	7.45 (s)	143.5 (d)
28	1.46 (s)	28.1 (q)	1.55 (s)	28.1 (q)
29	1.52 (s)	28.1 (q)	1.50 (s)	28.2 (q)
30	α 3.13 (dd, 19, 1.5)	38.0 (t)	α 3.27 (d, 16.5)	39.3 (t)
	β 2.92(m)^a^		β 2.91(m)^a^	

^a^Overlapped, without denoting multiplicity.
