# Supplementary material for: 16-nor Limonoids from Harrisonia perforata as promising selective 11β-HSD1 inhibitors
Source: Sci Rep. 2016 Nov 11;6:36927. doi: 10.1038/srep36927 (PMC5105127; doi:10.1038/srep36927)

Electronic Supplementary Information

16-nor Limonoids from *Harrisonia perforata* as promising selective 11*β*-HSD1 inhibitors

Xiaohui Yan[1,5,Δ], Ping Yi[2,Δ], Pei Cao[1], Shiying Yang[3], Xin Fang[1], Yu Zhang[1], Bin Wu[1], Ying Leng[4], Yingtong Di[1,*], Yang Lv[3,*], Xiaojiang Hao[1,*]

[1] State Key Laboratory of Phytochemistry and Plant Resources in West China, Kunming Institute of Botany, Chinese Academy of Sciences, Kunming 650204, P. R. China

[2] Key Laboratory of Chemistry for Natural Products of Guizhou Province and Chinese Academy of Sciences, Guiyan 550002, P. R. China

[3] Beijing Key Laboratory of Polymorphic Drugs, Institute of Materia Medica, Chinese Academy of Medical Sciences& Peking Union Medical College, Beijing 100050, P. R. China

[4] Shanghai Institute of Materia Medica, Chinese Academy of Sciences, Shanghai 200031, P. R. China

[5] College of Forestry, Southwest Forestry University/Key Laboratory of Forest Disaster Warning and Control of Yunnan Province, Kunming 650224, P. R. China

Correspondence and requests for materials should be addressed to Y. Di (email: diyt@mail.kib.ac.cn); Y. Lv (email: luy@imm.ac.cn); X. Hao (email: haoxj@mail.kib.ac.cn)

Contents

1. The Docking study of Harperspinoid A on murine 11*β*-HSD1 3
2.Supplementary Tables 4
    Table S1. Crystallographic Data for Polymorphs I and II of compound **1** 4
    Table S2. The dihedral angle of two polymorphs of compound **1**. 5
    Table S3. Molecular energy of the two crystal polymorphs of **1**. 5
    Table S4. Conformational analysis of **1** in the Gas Phase 5
    Table S5. The coordinate information of BL 6
    Table S6. The coordinate information of HCR. 8
    Table S7. The coordinate information of HCL 10
    Table S8. The coordinate information of BR. 12
    Table S9. The coordinate information of **TS1** (**HCR-BR**). 14
    Table S10. The coordinate information of **TS2** (**HCR-HCL**). 16
    Table S11. Inhibition of compound **1** on murine 11*β*-HSD1........ 18
3. Supplementary reference 19
    Full citation of Gaussian 03 19
4. Supplementary Figures 20
    Figure S1. IR spectrum of Harperspinoid A (**1**) 20
    Figure S2. CD spectrum of of Harperspinoid A (**1**) 20
    Figure S3. HRESIMS spectrum of of Harperspinoid A (**1**) 21
    Figure S4. ^{1}H NMR spectrum of Harperspinoid A (**1**) 22
    Figure S5. ^{13}C NMR spectrum of Harperspinoid A (**1**) 20
    Figure S6. ^{1}H-^{1}H COSY spectrum of of Harperspinoid A (**1**) 24
    Figure S7. HSQC spectrum of of Harperspinoid A (**1**) 25
    Figure S8. HMBC spectrum of Harperspinoid A (**1**) 26
    Figure S9. ROESY spectrum of Harperspinoid A (**1**) 27
    Figure S10. IR spectrum of Harperspinoid B (**2**) 28
    Figure S11. CD spectrum of of Harperspinoid B (**2**) 28
    Figure S12. HRESIMS spectrum of of Harperspinoid B (**2**) 29
    Figure S13. 1H NMR spectrum of Harperspinoid B (2) 30
    Figure S14. ^{13}C NMR spectrum of Harperspinoid B (**2**) 31
    Figure S15. ^{1}H-^{1}H COSY spectrum of of Harperspinoid B (**2**) 32
    Figure S16. HSQC spectrum of of Harperspinoid B (**2**) 33
    Figure S17. HMBC spectrum of Harperspinoid B (**2**) 34
    Figure S18. ROESY spectrum of Harperspinoid B (**2**) 35

The Docking study of Harperspinoid A on murine 11*β*-HSD1

The Docking study showed Harperspinoid A occupied the part NADPH site of 11*β*-HSD1 enzyme. Further analyses using LigPlot+ program revealed the Interactions of the ligand and the enzymes. The hydrophobic residues of Ala65, Ala42, Gly41, Lys44, His120, Tyr183, Ile121, Ala223, Thr220, Gly45, Leu215, Leu217 and Gly216 of 4K26 structure have hydrophobic interactions with the skeleton of Harperspinoid A. Furthermore, H-bonds with the residues of Ser43, Gly47, Asn119, Ile46 and Ile218 strengthened affinities of the enzyme. These interactions can explained that the compound showed significant inhibitory activity against murine 11*β*-HSD1 enzyme with IC_{50} value of 0.60 μM. Harperspinoid A showed low inhibitory activity against human 11*β*-HSD1 enzyme. We think there are two reasons that can explain the result： First, the active site residues of human 11*β*-HSD1 enzyme are different from the murine one. Second, less H-bonds are formed compared with the murine one means the compound has less affinities with the human enzyme.

1. Supplementary Tables

Crystallographic data for Harperspinoid D (**1**) have been deposited at the Cambridge Crystallographic Data center (deposition no. CDDD 999635 and 999636). Copies of data can be obtained free of charge via https://www.ccdc.cam.ac.uk/services/structure_deposit/.

Table S1. Crystallographic Data for Polymorphs I and II of compound **1**

		**HA（999636）**	**HB（999635）**
**colour/shape**		Colorless prism	Colorless needle
**crystal size (mm^3)**		0.10×0.10×0.30	0.05×0.10×0.40
**formula**		$C_{25}H_{28}O_7$	$C_{25}H_{28}O_7$
**crystal system**		monoclinic	orthorhombic
**space group**		$P2_1$	$P2_12_12_1$
	**a (Å)**	7.693(1)	10.310(1)
	**b (Å)**	7.727(1)	6.714(1)
**lattice**	**c (Å)**	37.712(1)	16.307(1)
**parameters**	**α (º)**	90.00	90.00
	**β (º)**	90.00	100.27(1)
	**γ (º)**	90.00	90.00
	**V ($Å^3$)**	1110.7(2)	2241.7(1)
	**Z**	2	4
**density ($_{calcd}$) (Mgm^{-3})**		1.317	1.305
**theta range for data collection (deg)**		2.75<θ<59.73	2.34<θ<58.76
**no. unique reflns**		2362	1997
**no. observed reflns $I > 2\sigma(I)$**		2303	1899
**completeness**		93.8％	64.9％
**Final R indices [$I > 2\sigma(I)$]**		R=0.0264	R=0.0286
		wR_2=0.0743	wR_2=0.0746
**Goodness-of fit, S**		S=1.027	S=1.028
**Solvent existence**		no	no
**Hydrogen bonds**		no	no
**Melting point(°C)**		195-197	176-177

Table S2. The dihedral angle of two polymorphs of compound **1.**

polymorphs	dihedral angle				
	A/B_1(°)	B_1/B_2(°)	B_2/C(°)	C/D(°)	D/E(°)
HA	21.6	104.3	15.6	22.7	100.5
HB	72.3	97.6	11.8	25.5	53.9

Table S3. Molecular energy of the two crystal polymorphs of **1.**

Energy (kcals/mol)	HA (**999635**)	HB (**999636**)
Bond Stretching Energy	187.081	185.494
Angle Bending Energy	44.734	47.650
Torsional Energy	25.419	23.406
Out of Plane Bending Energy	1.226	1.979
1-4 van der Waals Energy	8.753	8.434
van der Waals Energy	-7.113	-8.869
Total Energy	260.100	258.094

Table S4. Conformational analysis of **1** in the Gas Phase

**Species**	**ΔE**^{a}	**P**$_E$**%**^{b}	**Δ*E′***^{a}	**P**$_{E'}$**%**^{b}	**ΔG**^{a}	**P**$_G$**%**^{b}
**HCR**	0.00	42.8	0.00	44.1	0.00	47.6
**BL**	0.81	10.9	0.88	10.0	1.11	7.40
**HCL**	0.19	31.0	0.22	30.6	0.30	28.6
**BR**	0.61	15.3	0.63	15.3	0.63	16.4

^{a} Relative energy, relative zero point energy, and relative Gibbs free energy at the B3LYP/6-31G* level, respectively (kcal/mol). ^{b} Conformational distribution calculated by using the respective parameters above at the B3LYP/6-31G* level.

Table S5. The coordinate information of HCR.

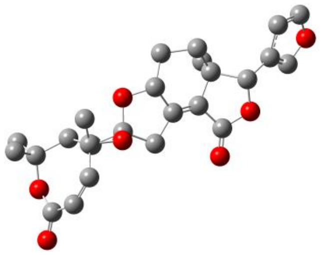

Free Energy= -1495.607160 a. u.

	X	Y	Z
O	-5.46096	0.80321	1.05052
O	-6.30846	2.63504	0.16649
O	-2.0951	-0.14684	-1.30589
O	-0.75976	-1.25083	0.27133
O	2.29315	3.02527	0.12102
O	4.13397	1.77665	-0.31209
O	7.97024	0.38071	0.45015
C	-4.30667	0.40447	-1.83526
H	-4.07483	0.45762	-2.89901
C	-5.22253	1.27509	-1.38999
H	-5.65946	1.96259	-2.10901
C	-5.70857	1.59747	-0.02211
C	-5.05459	-0.59463	1.01217
C	-3.64565	-0.77366	0.4066
H	-3.34348	-1.79709	0.65735
C	-2.56057	0.16798	0.95934
H	-2.23081	-0.08417	1.96889
H	-2.90143	1.20794	0.94829
C	-1.43924	-0.02385	-0.05116
C	0.93248	0.24768	-0.1623
C	0.52286	-1.19134	-0.36103
H	0.38924	-1.35698	-1.44519
C	-3.46304	-0.63429	-1.13257
C	1.53274	-2.19755	0.17276
H	1.28686	-3.2075	-0.17254
H	1.48478	-2.20893	1.26707
C	2.94866	-1.79505	-0.31256
H	3.67748	-2.55048	0.00307
H	2.95582	-1.78395	-1.41192
C	3.34136	-0.40508	0.22337
C	2.20564	0.58521	0.05559
C	2.80077	1.93828	-0.01023
C	4.42779	0.37397	-0.58495
H	4.24394	0.20729	-1.65659

C	3.74166	-0.48158	1.71555
H	2.91507	-0.8663	2.31977
H	4.00786	0.50483	2.1081
H	4.60779	-1.13812	1.84755
C	-3.55595	-1.97429	-1.87439
H	-3.30002	-1.83945	-2.93041
H	-4.56156	-2.40247	-1.81686
H	-2.84292	-2.67966	-1.43687
C	5.86526	0.11477	-0.27326
C	6.73979	0.95847	0.34407
H	6.64609	1.96106	0.72882
C	6.61741	-1.07627	-0.57501
H	6.26353	-1.96748	-1.07531
C	7.87713	-0.8562	-0.11185
H	8.78195	-1.44463	-0.10661
C	-6.14135	-1.43117	0.31978
H	-6.27442	-1.16183	-0.72981
H	-7.09571	-1.2799	0.83376
H	-5.89061	-2.49617	0.37332
C	-5.01933	-0.96394	2.50376
H	-6.011	-0.82077	2.9423
H	-4.31563	-0.33082	3.05031
H	-4.72655	-2.01093	2.63466
C	-0.33361	1.05904	-0.17794
H	-0.47203	1.62511	-1.1047
H	-0.35925	1.77425	0.64878

Table S6. The coordinate information of **BL**

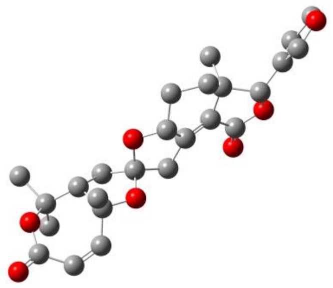

Free Energies= -1495.605393 a.u.

	X	Y	Z
O	-6.01694	-0.45679	0.61507
O	-7.65234	-0.73918	-0.85737
O	-2.06443	-0.12608	-1.16106
O	-0.73432	-0.81254	0.6533
O	2.58103	2.99279	-0.80477
O	4.32547	1.54595	-0.84939
O	7.92507	-1.20603	-0.18046
C	-4.32656	0.05058	-1.80592
H	-3.81762	0.37134	-2.7129
C	-5.66017	0.14427	-1.7458
H	-6.21988	0.51307	-2.60036
C	-6.52252	-0.35605	-0.64718
C	-4.96784	0.41443	1.15637
C	-3.57548	-0.09592	0.70458
H	-3.36017	-0.97764	1.31712
C	-2.40778	0.88845	0.8971
H	-2.66481	1.89045	0.53947
H	-2.06047	0.96067	1.93061
C	-1.34191	0.30391	-0.02096
C	1.03766	0.35795	-0.23123
C	0.5308	-1.04071	0.02242
H	0.3546	-1.51941	-0.9575
C	-3.39935	-0.56543	-0.7843
C	1.4875	-1.90548	0.83196
H	1.16921	-2.95316	0.80957
H	1.46413	-1.5827	1.87852
C	2.91379	-1.76509	0.24191
H	2.8938	-2.08577	-0.80949
H	3.60429	-2.43665	0.76492
C	3.40553	-0.30828	0.33038
C	2.33359	0.65997	-0.12588
C	3.015	1.88415	-0.60378
C	4.51648	0.10574	-0.68638

H	4.28306	-0.36333	-1.652
C	3.83225	0.03799	1.77598
H	4.13905	1.08507	1.86469
H	4.67432	-0.58753	2.08925
H	3.00292	-0.12008	2.4714
C	-3.45476	-2.09969	-0.89928
H	-3.27356	-2.41245	-1.93246
H	-4.43484	-2.4735	-0.58716
H	-2.68212	-2.53606	-0.25929
C	5.94476	-0.16253	-0.33799
C	6.66279	-1.2699	-0.68574
H	6.43295	-2.15022	-1.26731
C	6.84248	0.65482	0.43572
H	6.6295	1.62635	0.85674
C	8.01815	-0.02391	0.49497
H	8.97699	0.18307	0.94495
C	-5.27233	1.8893	0.84519
H	-6.33076	2.08332	1.04708
H	-5.06023	2.1717	-0.18707
H	-4.68617	2.53803	1.50361
C	-5.11449	0.19045	2.66833
H	-5.04814	-0.87622	2.90216
H	-6.08563	0.55721	3.01379
H	-4.32675	0.71912	3.21572
C	-0.17301	1.2087	-0.49735
H	-0.3023	1.45115	-1.55734
H	-0.12741	2.15288	0.05196

Table S7. The coordinate information of **HCL**

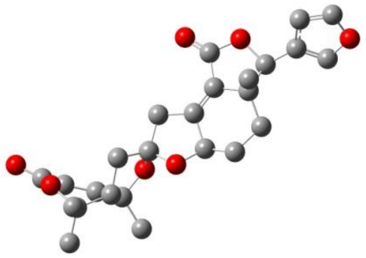

Free Energies = -1495.606152 a.u.

Center Number	Atomic Number	Atomic Type	Coordinates (Angstroms) X	Y	Z
1	8	0	-5.471582	0.810282	1.020595
2	8	0	-6.320799	2.620122	0.093932
3	8	0	-2.087022	-0.173459	-1.298264
4	8	0	-0.755749	-1.235377	0.310892
5	8	0	2.288938	3.048471	0.070339
6	8	0	4.131412	1.792286	-0.335909
7	8	0	7.915008	-0.788318	-0.367732
8	6	0	-4.298100	0.357443	-1.850086
9	1	0	-4.060592	0.389588	-2.913446
10	6	0	-5.220102	1.233073	-1.427896
11	1	0	-5.655942	1.903740	-2.163307
12	6	0	-5.715567	1.581365	-0.069618
13	6	0	-5.056901	-0.585571	1.013671
14	6	0	-3.643952	-0.769442	0.419294
15	1	0	-3.337541	-1.785536	0.693831
16	6	0	-2.566753	0.190014	0.956754
17	1	0	-2.241494	-0.037696	1.973557
18	1	0	-2.912570	1.227781	0.920372
19	6	0	-1.438909	-0.019146	-0.043076
20	6	0	0.932219	0.260228	-0.150436
21	6	0	0.528965	-1.184792	-0.318104
22	1	0	0.399249	-1.374273	-1.398872
23	6	0	-3.454035	-0.662720	-1.121405
24	6	0	1.540976	-2.176392	0.239373
25	1	0	1.299183	-3.194100	-0.085470
26	1	0	1.489743	-2.165615	1.333564
27	6	0	2.956132	-1.778640	-0.251652
28	1	0	3.691105	-2.521818	0.078410
29	1	0	2.963910	-1.790651	-1.351046

30	6	0	3.340715	-0.375662	0.253767
31	6	0	2.203608	0.607547	0.062423
32	6	0	2.798476	1.959489	-0.036454
33	6	0	4.423793	0.381513	-0.578268
34	1	0	4.229849	0.187696	-1.642188
35	6	0	3.735921	-0.421283	1.748219
36	1	0	2.916016	-0.819366	2.352807
37	1	0	3.970533	0.576661	2.132109
38	1	0	4.617176	-1.054232	1.894155
39	6	0	-3.537574	-2.018652	-1.834723
40	1	0	-3.274969	-1.905622	-2.891672
41	1	0	-4.542166	-2.448914	-1.774728
42	1	0	-2.825252	-2.712081	-1.377319
43	6	0	5.865884	0.122737	-0.282366
44	6	0	6.662846	-0.795795	-0.901874
45	1	0	6.499674	-1.495835	-1.707697
46	6	0	6.699036	0.754747	0.707107
47	1	0	6.414710	1.556622	1.372496
48	6	0	7.919756	0.166273	0.607556
49	1	0	8.858939	0.305638	1.120643
50	6	0	-6.135402	-1.442482	0.333307
51	1	0	-6.264839	-1.194965	-0.722145
52	1	0	-7.093122	-1.286523	0.839589
53	1	0	-5.878768	-2.504703	0.409388
54	6	0	-5.027064	-0.923083	2.512898
55	1	0	-6.021728	-0.776391	2.943423
56	1	0	-4.329668	-0.274373	3.049192
57	1	0	-4.728794	-1.965307	2.667382
58	6	0	-0.337329	1.065685	-0.188422
59	1	0	-0.474645	1.611936	-1.127162
60	1	0	-0.369478	1.797554	0.623380

Table S8. The coordinate information of **BR.**

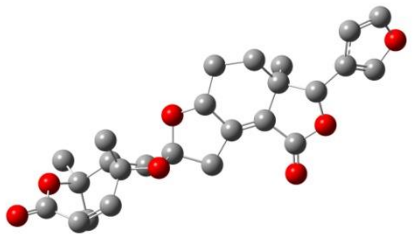

Free Energies= -1495.606677 a.u.

O	-6.02167	-0.48207	0.59338
O	-7.67225	-0.60219	-0.88424
O	-2.0788	-0.02093	-1.17661
O	-0.73311	-0.90512	0.53802
O	2.57514	3.02928	-0.50802
O	4.32559	1.60228	-0.6949
O	8.07887	0.13936	0.31148
C	-4.34325	0.23227	-1.78399
H	-3.83818	0.62999	-2.66219
C	-5.67513	0.33481	-1.70352
H	-6.2367	0.78784	-2.51528
C	-6.53558	-0.2556	-0.64906
C	-4.95619	0.32008	1.20457
C	-3.57543	-0.16758	0.69613
H	-3.37461	-1.1104	1.21539
C	-2.39064	0.77301	0.98149
H	-2.63622	1.81165	0.73921
H	-2.03239	0.72673	2.01272
C	-1.34055	0.28065	-0.00634
C	1.03773	0.34788	-0.22538
C	0.52759	-1.06826	-0.12116
H	0.34544	-1.43895	-1.14577
C	-3.41398	-0.48665	-0.83411
C	1.48548	-2.01578	0.58791
H	1.16694	-3.05486	0.45284
H	1.46397	-1.80763	1.66308
C	2.91213	-1.81303	0.01672
H	2.89328	-2.01561	-1.06368
H	3.59831	-2.54057	0.46551
C	3.40611	-0.37507	0.26655
C	2.33365	0.63583	-0.08692
C	3.01175	1.9069	-0.42416

C	4.52561	0.15651	-0.68496
H	4.31357	-0.2055	-1.70166
C	3.82785	-0.18476	1.74281
H	4.16917	0.83801	1.93107
H	4.64839	-0.8629	1.99838
H	2.98832	-0.38295	2.4154
C	-3.48299	-2.00178	-1.10015
H	-3.31214	-2.21177	-2.16072
H	-4.4641	-2.39686	-0.81885
H	-2.70967	-2.50616	-0.51324
C	5.9489	-0.12861	-0.33341
C	6.8862	0.7634	0.09487
H	6.86291	1.82715	0.26759
C	6.61798	-1.40316	-0.38891
H	6.19923	-2.35203	-0.69548
C	7.89712	-1.17714	0.01423
H	8.76248	-1.81068	0.13559
C	-5.23878	1.82227	1.03183
H	-6.29478	2.01222	1.24968
H	-5.01982	2.19583	0.03063
H	-4.64534	2.39868	1.74849
C	-5.09666	-0.03892	2.69059
H	-5.04003	-1.123	2.82653
H	-6.06205	0.30495	3.07374
H	-4.30043	0.43077	3.27793
C	-0.17092	1.2246	-0.39766
H	-0.30524	1.56966	-1.42825
H	-0.1183	2.11052	0.24067

Table S9. The coordinate information of **TS1 (HCR-BR)**.

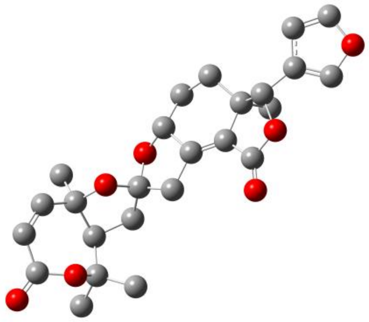

Free Energy= -1495.578982 a.u.

	X	Y	Z
O	5.028105	-1.243067	-0.724115
O	7.183045	-0.795877	-1.264255
O	2.238444	0.810910	-0.669856
O	0.634000	1.518473	0.901270
O	-1.977111	-2.870843	-0.277753
O	-3.880422	-1.711493	-0.688483
O	-7.881699	-0.751238	-0.113646
C	4.397656	1.404980	-1.469146
H	4.095821	2.280540	-2.042719
C	5.499026	0.767233	-1.878428
H	6.078015	1.153234	-2.713443
C	6.023046	-0.454537	-1.241177
C	4.598808	-1.140924	0.657068
C	3.883317	0.242755	0.944574
H	4.537643	0.780453	1.638079
C	2.471768	0.125689	1.585558
H	2.336531	0.876616	2.367034
H	2.280506	-0.841947	2.045711
C	1.460958	0.429870	0.456940
C	-0.858517	0.012418	0.021215
C	-0.563662	1.488453	0.118389
H	-0.332023	1.857067	-0.896968
C	3.544364	1.236307	-0.225203
C	-1.708353	2.308560	0.697739
H	-1.526203	3.378408	0.550359
H	-1.762102	2.135476	1.778050
C	-3.030717	1.888772	0.006410
H	-3.852336	2.521858	0.360846
H	-2.935794	2.063500	-1.074859
C	-3.340565	0.402622	0.266522
C	-2.108506	-0.454552	0.055321
C	-2.568739	-1.818775	-0.285706
C	-4.275546	-0.308849	-0.763271

H	-4.012912	0.047524	-1.770172
C	-3.876234	0.192389	1.702409
H	-3.139942	0.516839	2.443263
H	-4.096926	-0.862342	1.894253
H	-4.800037	0.759840	1.854749
C	3.471526	2.672927	0.367810
H	2.998741	3.343047	-0.356429
H	4.480578	3.044834	0.579027
H	2.882467	2.714345	1.285102
C	-5.752579	-0.213248	-0.563490
C	-6.600032	-1.211126	-0.184397
H	-6.451910	-2.254838	0.040899
C	-6.577886	0.953436	-0.744773
H	-6.260624	1.939766	-1.055051
C	-7.849304	0.566625	-0.455543
H	-8.799815	1.077940	-0.448544
C	5.813114	-1.278493	1.588808
H	6.520104	-0.453461	1.454209
H	6.344002	-2.213365	1.388750
H	5.483884	-1.275520	2.634477
C	3.712062	-2.385511	0.793314
H	4.291983	-3.252933	0.466958
H	2.824978	-2.317222	0.158033
H	3.402268	-2.557534	1.827878
C	0.472851	-0.683390	-0.008909
H	0.750720	-1.021005	-1.012909
H	0.480340	-1.557836	0.645906

Table S10. The coordinate information of **TS2 (HCR-HCL)**.

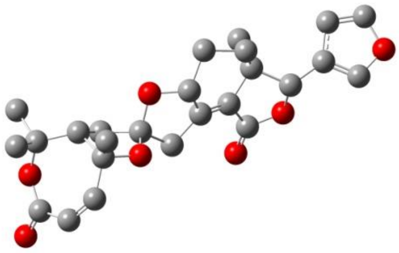

Free Energies= -1495.597737 a. u.

Center Number	Atomic Number	Atomic Type	Coordinates (Angstroms) X	Y	Z
1	8	0	-5.463259	0.686151	1.103788
2	8	0	-6.282005	2.629647	0.465214
3	8	0	-2.087812	0.008208	-1.335342
4	8	0	-0.772665	-1.294704	0.100506
5	8	0	2.274433	2.940805	0.640617
6	8	0	4.140764	1.751850	0.141915
7	8	0	8.088974	0.340224	-0.627031
8	6	0	-4.286624	0.659653	-1.800514
9	1	0	-4.043627	0.851814	-2.845578
10	6	0	-5.194200	1.476742	-1.249333
11	1	0	-5.614145	2.260947	-1.873211
12	6	0	-5.691264	1.619501	0.144832
13	6	0	-5.074574	-0.699203	0.881450
14	6	0	-3.661866	-0.814736	0.268592
15	1	0	-3.373137	-1.865610	0.385597
16	6	0	-2.572983	0.034993	0.948374
17	1	0	-2.254473	-0.351266	1.918327
18	1	0	-2.905627	1.070196	1.072770
19	6	0	-1.443465	-0.032757	-0.069344
20	6	0	0.931133	0.238850	-0.096230
21	6	0	0.524701	-1.161457	-0.488964
22	1	0	0.418458	-1.190003	-1.588288
23	6	0	-3.463771	-0.476378	-1.237185
24	6	0	1.516229	-2.233911	-0.057435
25	1	0	1.281330	-3.188608	-0.540132
26	1	0	1.429875	-2.388188	1.023836
27	6	0	2.947712	-1.779014	-0.436267
28	1	0	3.666791	-2.572914	-0.204692
29	1	0	2.994093	-1.618583	-1.522614
30	6	0	3.322726	-0.476292	0.294912

31	6	0	2.194450	0.538907	0.211441
32	6	0	2.794628	1.882544	0.378488
33	6	0	4.422623	0.397660	-0.370529
34	1	0	4.195228	0.437571	-1.444511
35	6	0	3.608743	-0.739836	1.797469
36	1	0	2.678617	-0.960562	2.328494
37	1	0	4.062781	0.133108	2.276070
38	1	0	4.276959	-1.594394	1.935514
39	6	0	-3.570337	-1.704996	-2.150327
40	1	0	-3.303098	-1.436307	-3.177547
41	1	0	-4.582793	-2.120505	-2.156189
42	1	0	-2.872167	-2.473602	-1.804930
43	6	0	5.889158	0.114112	-0.218737
44	6	0	6.843039	0.776655	-0.940342
45	1	0	6.788815	1.567750	-1.673194
46	6	0	6.622080	-0.816328	0.603892
47	1	0	6.231708	-1.529114	1.312268
48	6	0	7.937553	-0.635519	0.312677
49	1	0	8.848176	-1.094890	0.665095
50	6	0	-6.165827	-1.419470	0.074746
51	1	0	-6.284586	-1.010678	-0.930540
52	1	0	-7.122815	-1.323246	0.597018
53	1	0	-5.930871	-2.485870	-0.011986
54	6	0	-5.058256	-1.266366	2.310033
55	1	0	-6.052329	-1.170755	2.756091
56	1	0	-4.352368	-0.721879	2.942510
57	1	0	-4.778905	-2.325138	2.300858
58	6	0	-0.332560	1.052233	-0.045996
59	1	0	-0.448767	1.718656	-0.906872
60	1	0	-0.374864	1.671085	0.854292

Table S11. Inhibition of compound **1** on murine 11*β*-HSD1

Compound	Concentration of	Inhibition rate	SD	IC_{50}
**1**	0.1 μM	22.78%	2.13%	0.6 μM
	0.3 μM	35.13%	1.65%	
	1 μM	55.75%	4.42%	
	3 μM	79.41%	2.17%	
	10 μM	90.52%	0.03%	
Glycyrrhizic acid	1 nM	21.24%	3.89%	37.3 nM
	10 nM	51.94%	6.22%	
	100 nM	86.85%	2.03%	

3. Supplementary reference

Full citation of Gaussian 03:

Frisch, M. J.; Trucks, G. W.; Schlegel, H. B.; Scuseria, G. E.; Robb, M. A.; Cheeseman, J. R.; Montgomery, J. A., Jr.; Vreven, T.; Kudin, K. N.; Burant, J. C.; Millam, J. M.; Iyengar, S. S.; Tomasi, J.; Barone, V.; Mennucci, B.; Cossi, M.; Scalmani, G.; Rega, N.; Petersson, G. A.; Nakatsuji, H.; Hada, M.; Ehara, M.; Toyota, K.; Fukuda, R.; Hasegawa, J.; Ishida, M.; Nakajima, T.; Honda, Y.; Kitao, O.; Nakai, H.; Klene, M.; Li, X.; Knox, J. E.; Hratchian, H. P.; Cross, J. B.; Bakken, V.; Adamo, C.; Jaramillo, J.; Gomperts, R.; Stratmann, R. E.; Yazyev, O.; Austin, A. J.; Cammi, R.; Pomelli, C.; Ochterski, J. W.; Ayala, P. Y.; Morokuma, K.; Voth, G. A.; Salvador, P.; Dannenberg, J. J.; Zakrzewski, V. G.; Dapprich, S.; Daniels, A. D.; Strain, M. C.; Farkas, O.; Malick, D. K.; Rabuck, A. D.; Raghavachari, K.; Foresman, J. B.; Ortiz, J. V.; Cui, Q.; Baboul, A. G.; Clifford, S.; Cioslowski, J.; Stefanov, B. B.; Liu, G.; Liashenko, A.; Piskorz, P.; Komaromi, I.; Martin, R. L.; Fox, D. J.; Keith, T.; Al-Laham, M. A.; Peng, C. Y.; Nanayakkara, A.; Challacombe, M.; Gill, P. M. W.; Johnson, B.; Chen, W.; Wong, M. W.; Gonzalez, C.; Pople, J. A. Gaussian03; D 01, Gaussian, Inc.: Wallingford, CT, 2005.

4. Supplementary Figures

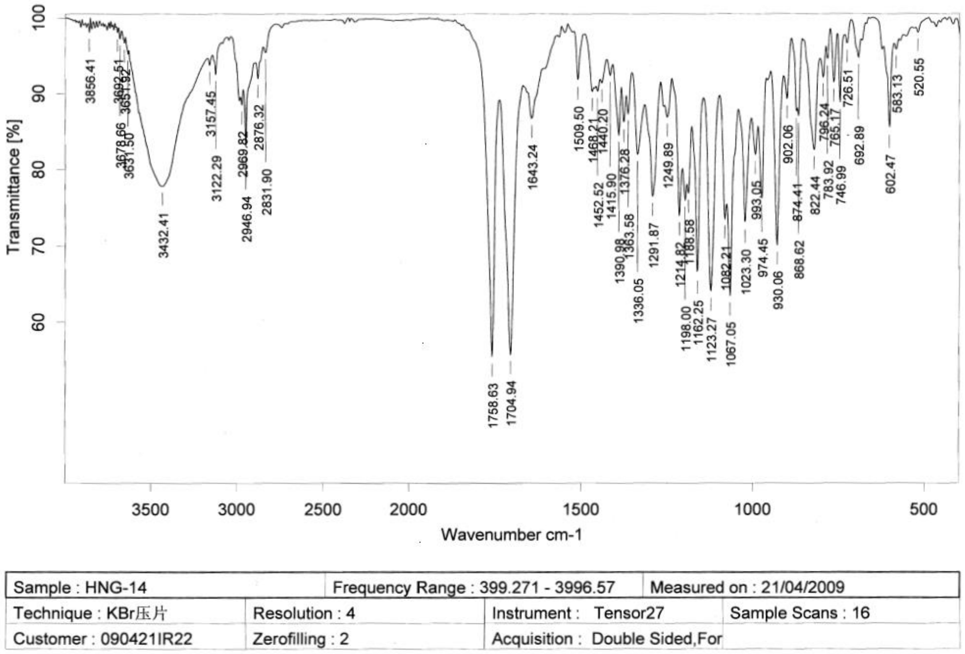

Figure S1. IR spectrum of Harperspinoid A (**1**)

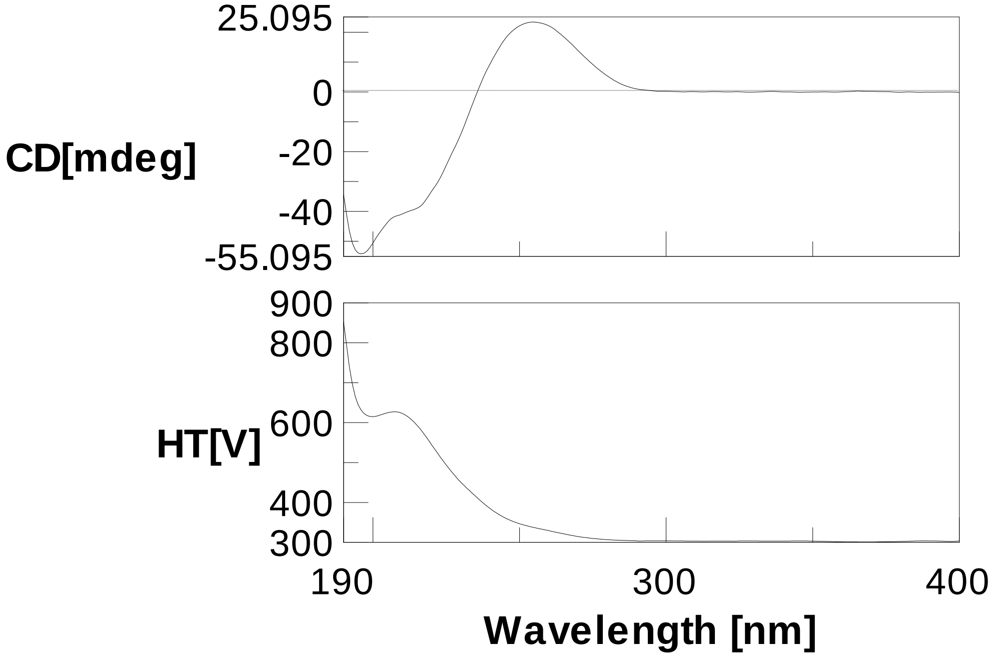

Figure S2. CD spectrum of Harperspinoid A (**1**)

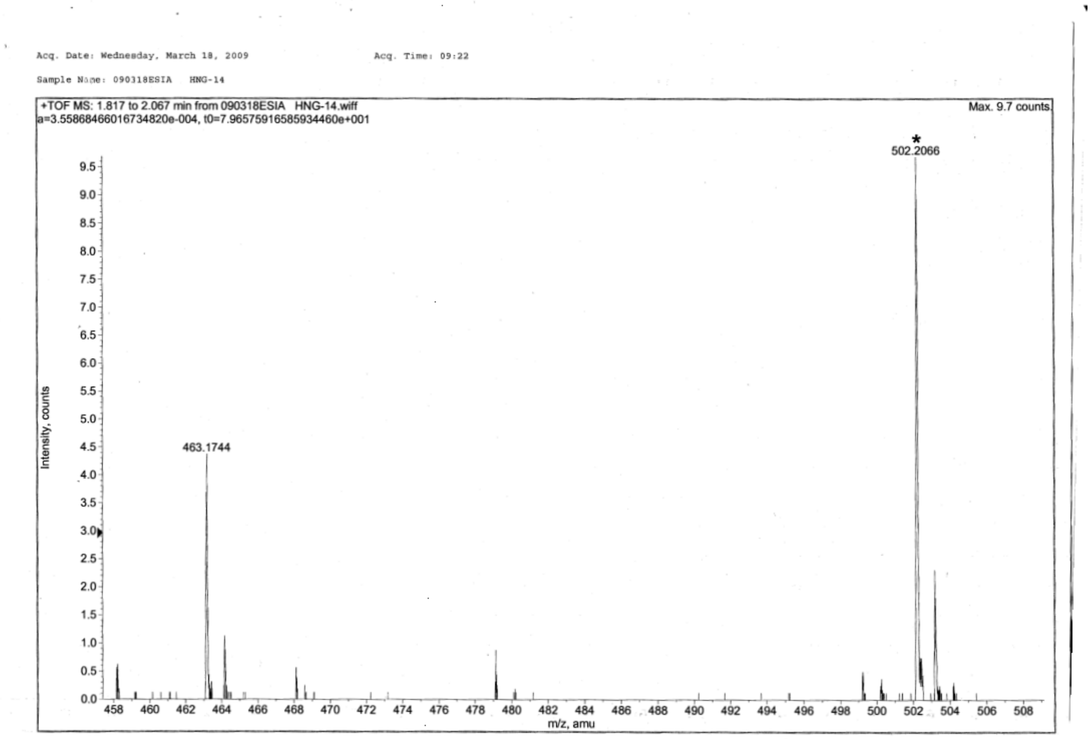

Figure S3. HRESIMS spectrum of Harperspinoid A (**1**)

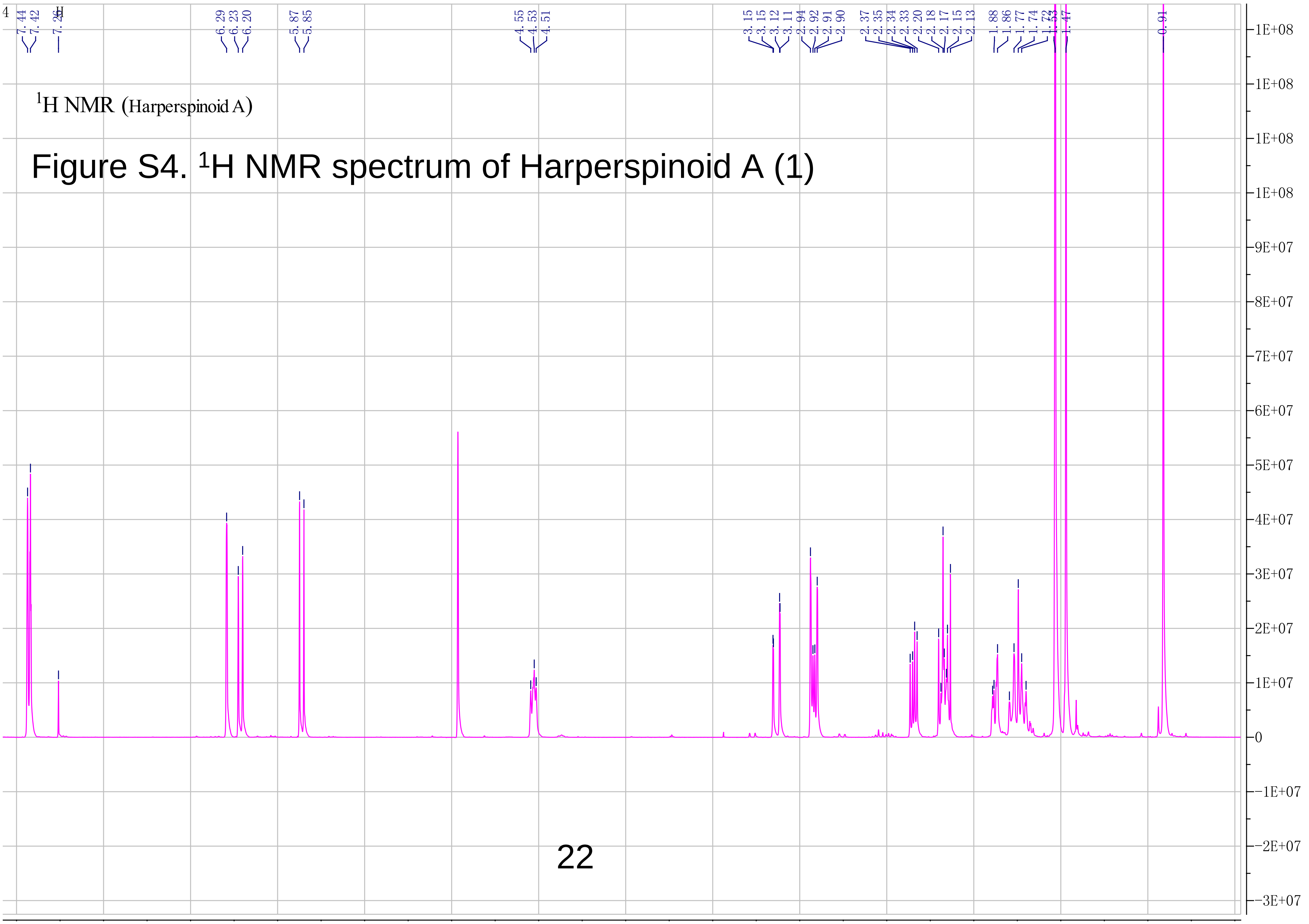

Figure S4. ^{1}H NMR spectrum of Harperspinoid A (1)

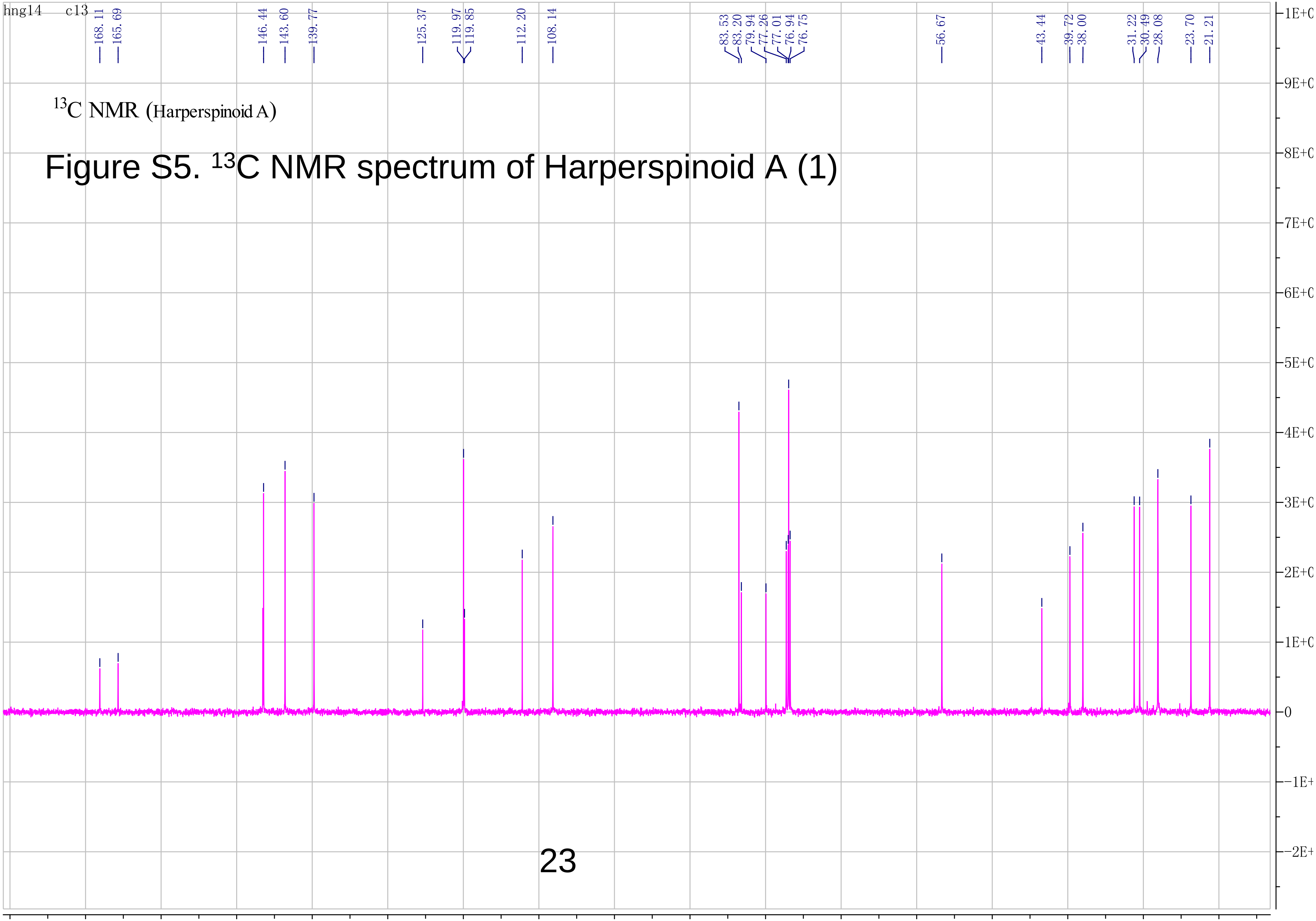

Figure S5. ^{13}C NMR spectrum of Harperspinoid A (1)

Figure S6. ^{1}H-^{1}H COSY spectrum of of Harperspinoid A (1)

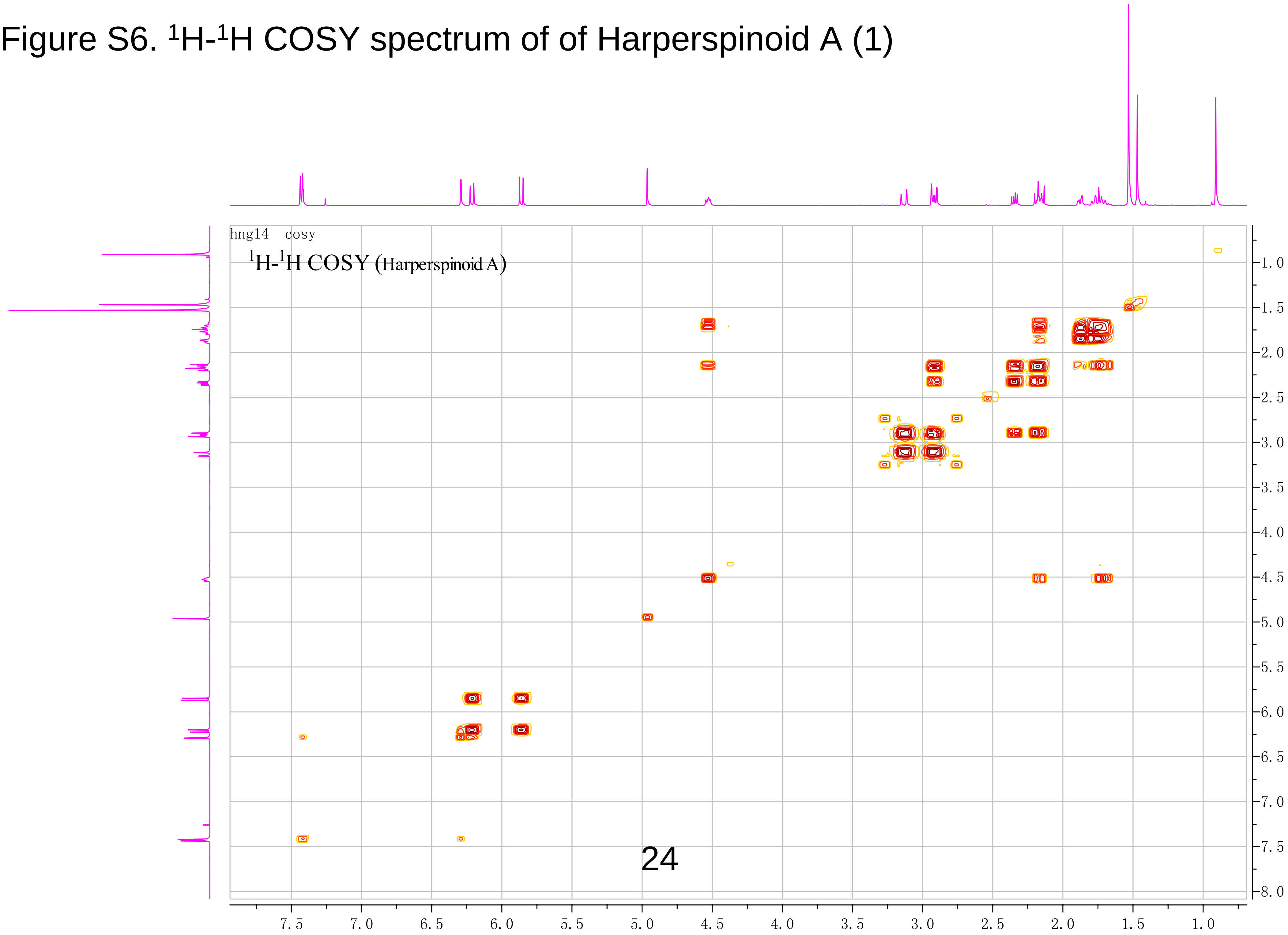

Figure S7. HSQC spectrum of of Harperspinoid A (1)

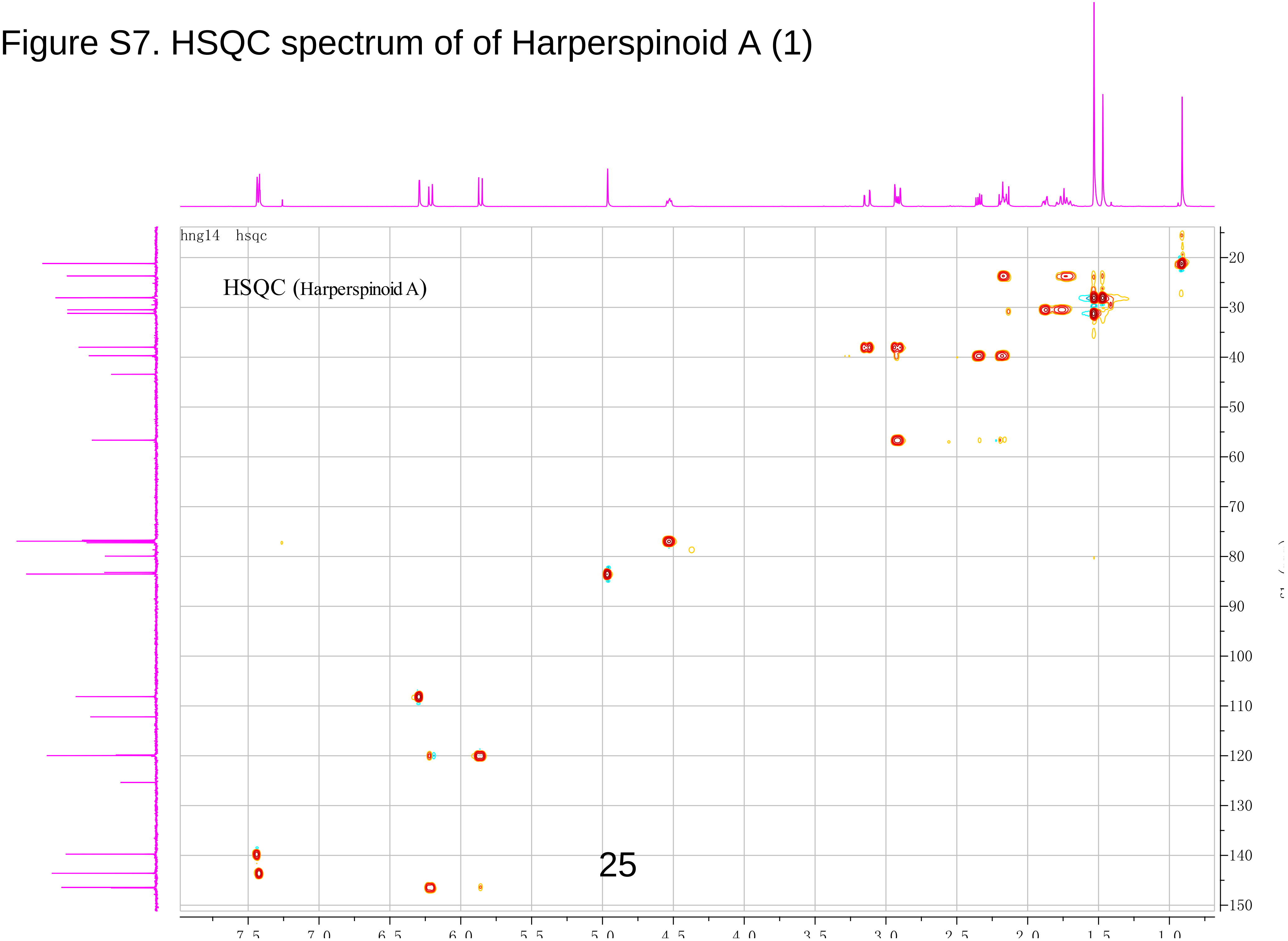

Figure S8. HMBC spectrum of Harperspinoid A (1)

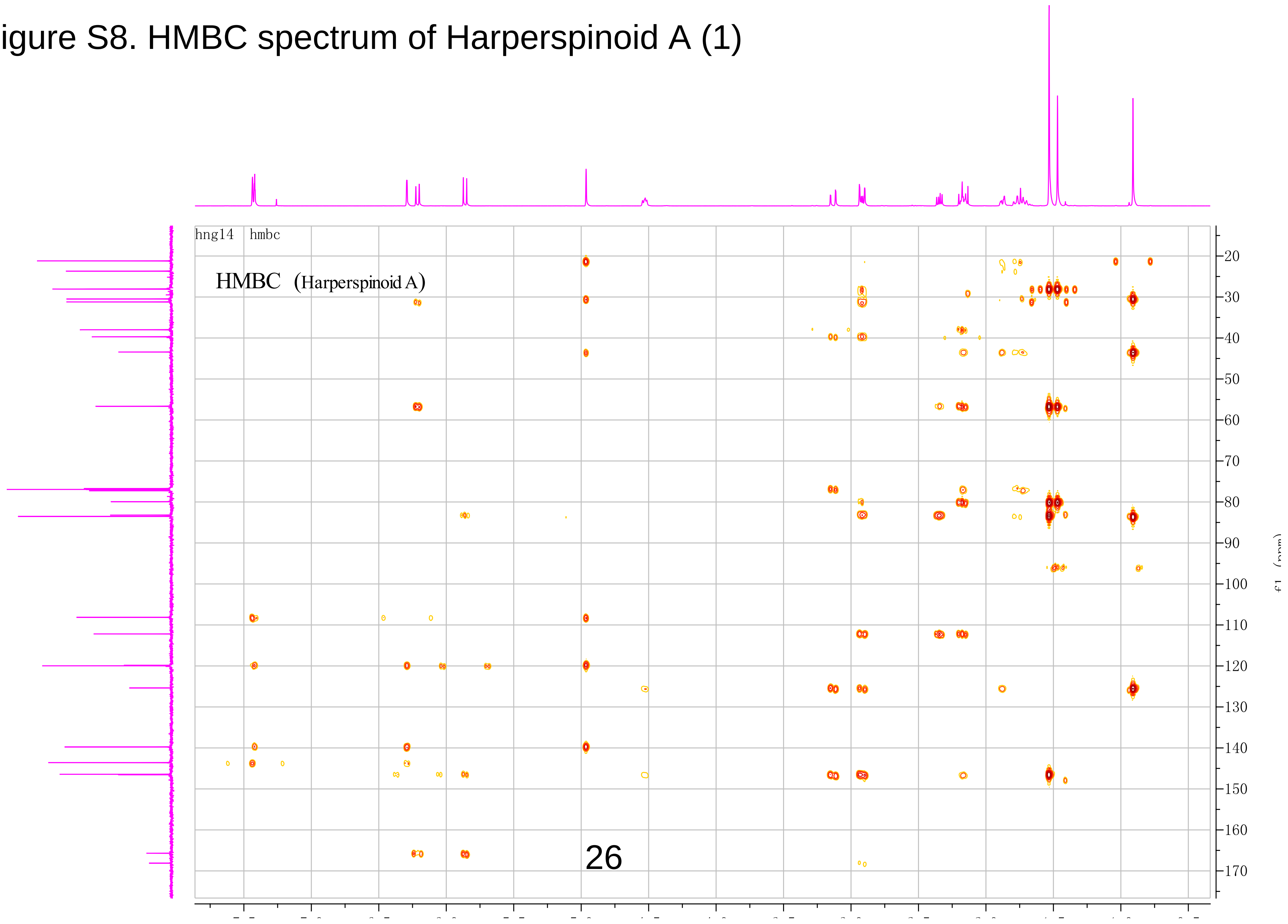

Figure S9. ROESY spectrum of Harperspinoid A (1)

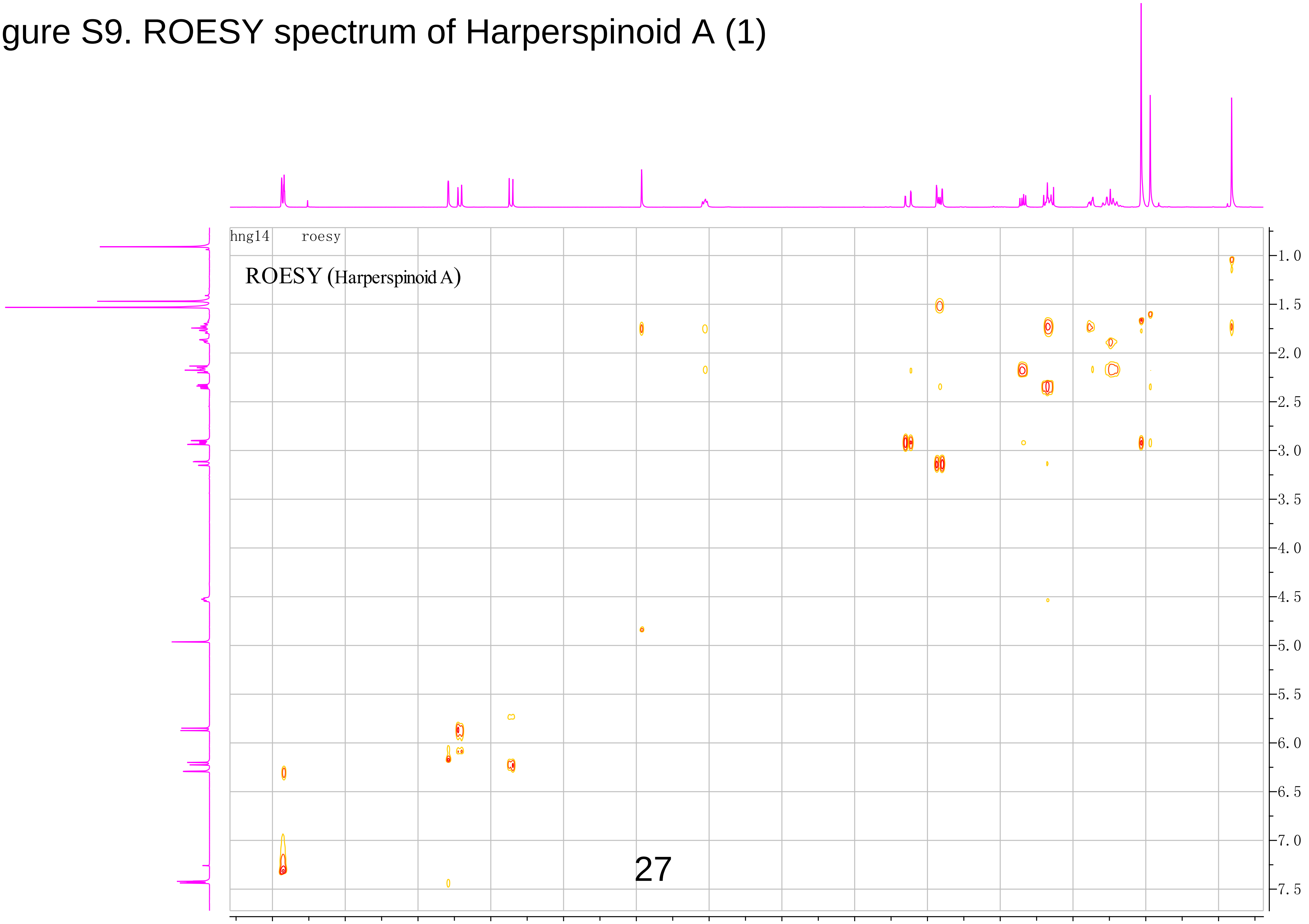

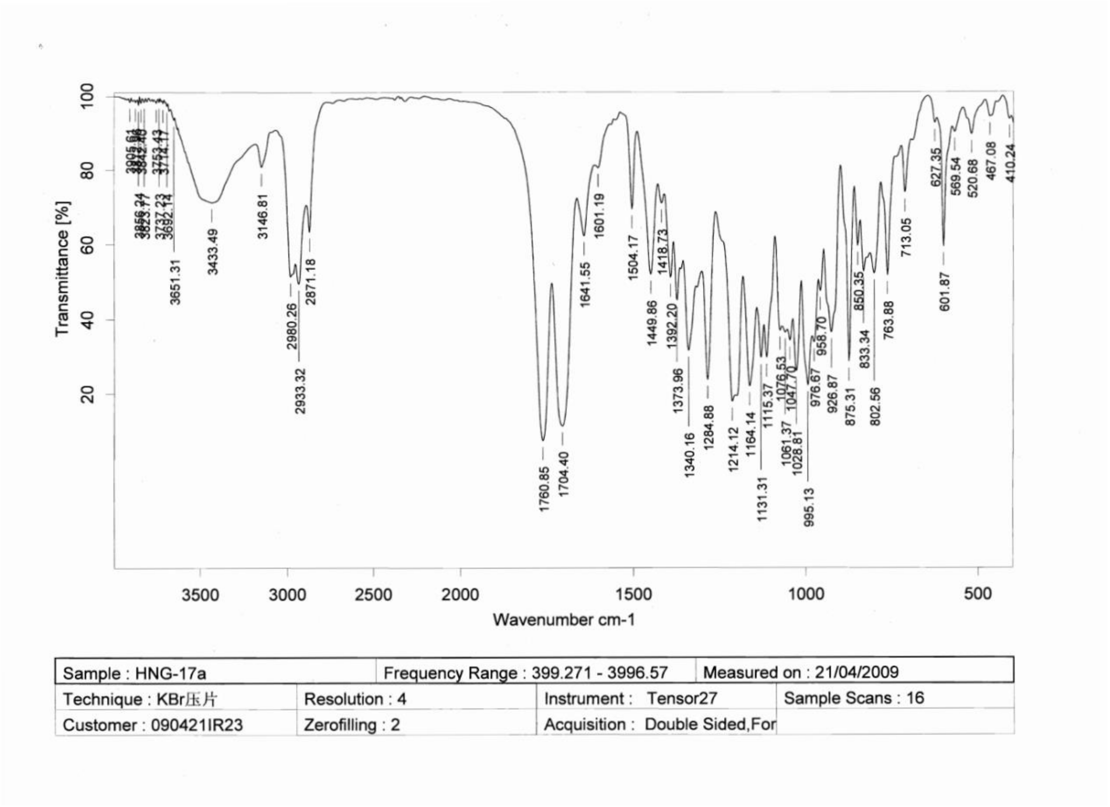

Figure S10. IR spectrum of Harperspinoid B (**2**)

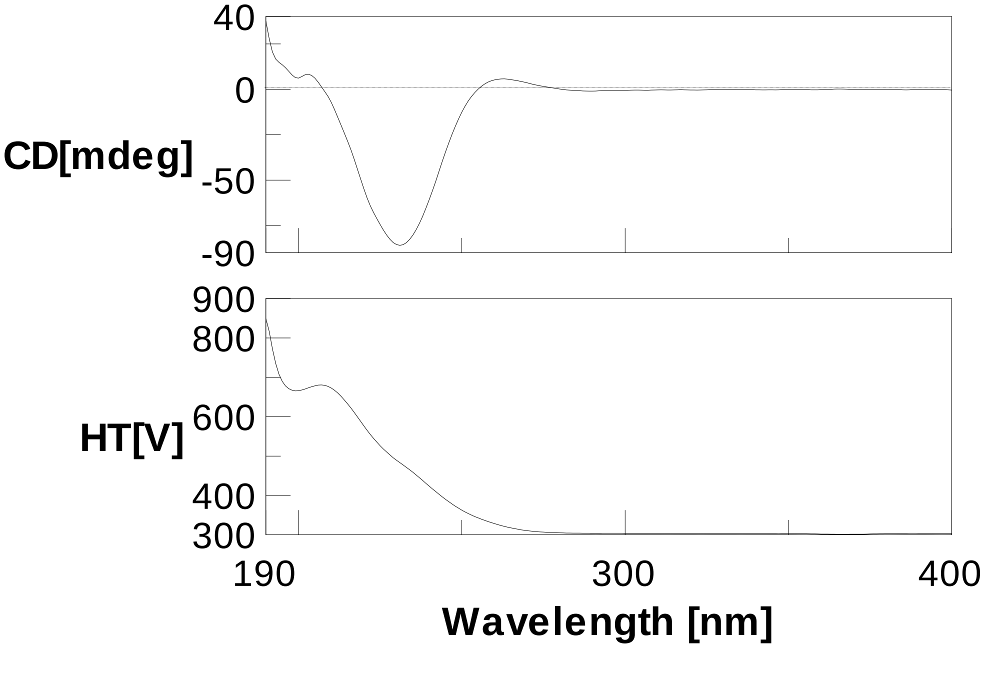

Figure S11. CD spectrum of Harperspinoid B (**2**)

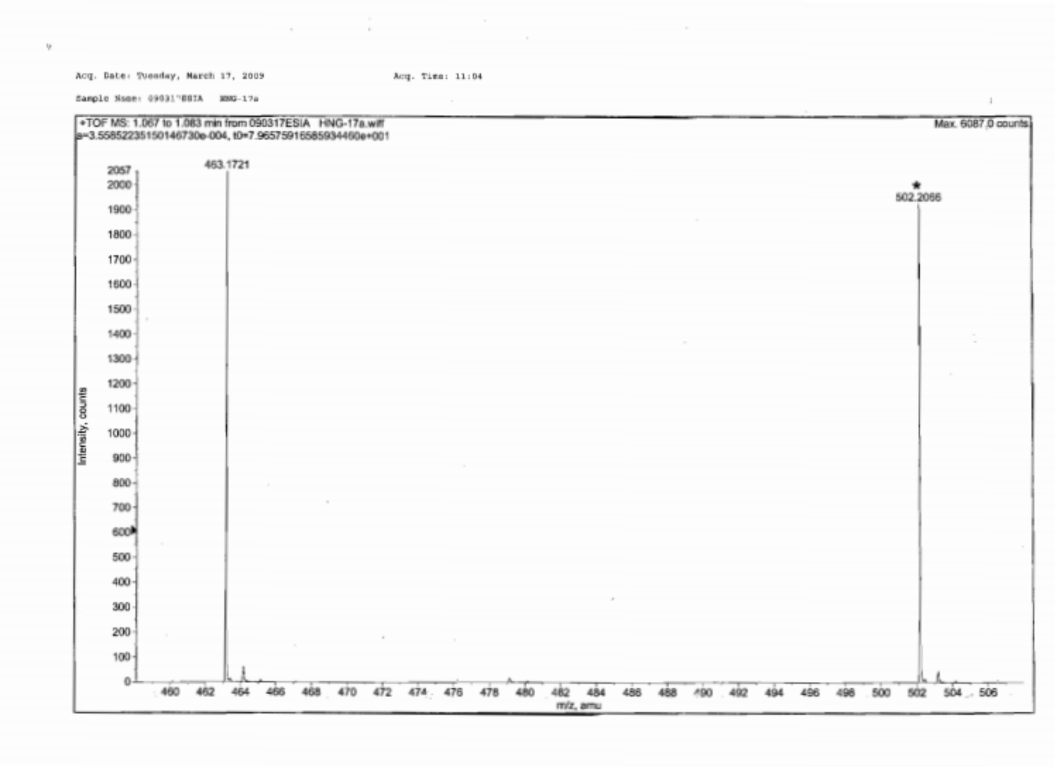

Figure S12. HRESIMS spectrum of Harperspinoid B (**2**)

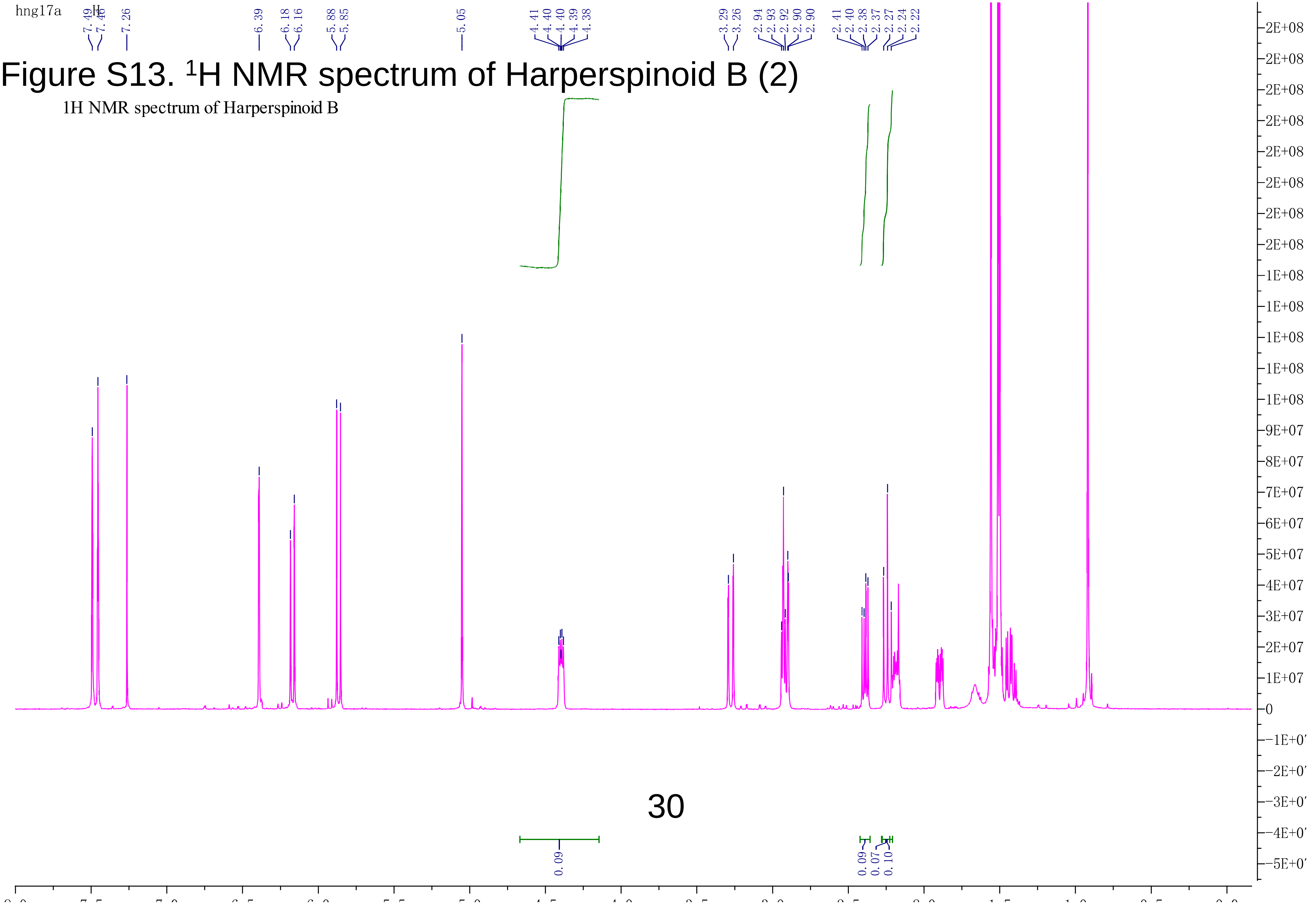

Figure S13. ^{1}H NMR spectrum of Harperspinoid B (2)

Figure S14. ^{13}C NMR spectrum of Harperspinoid B (2)

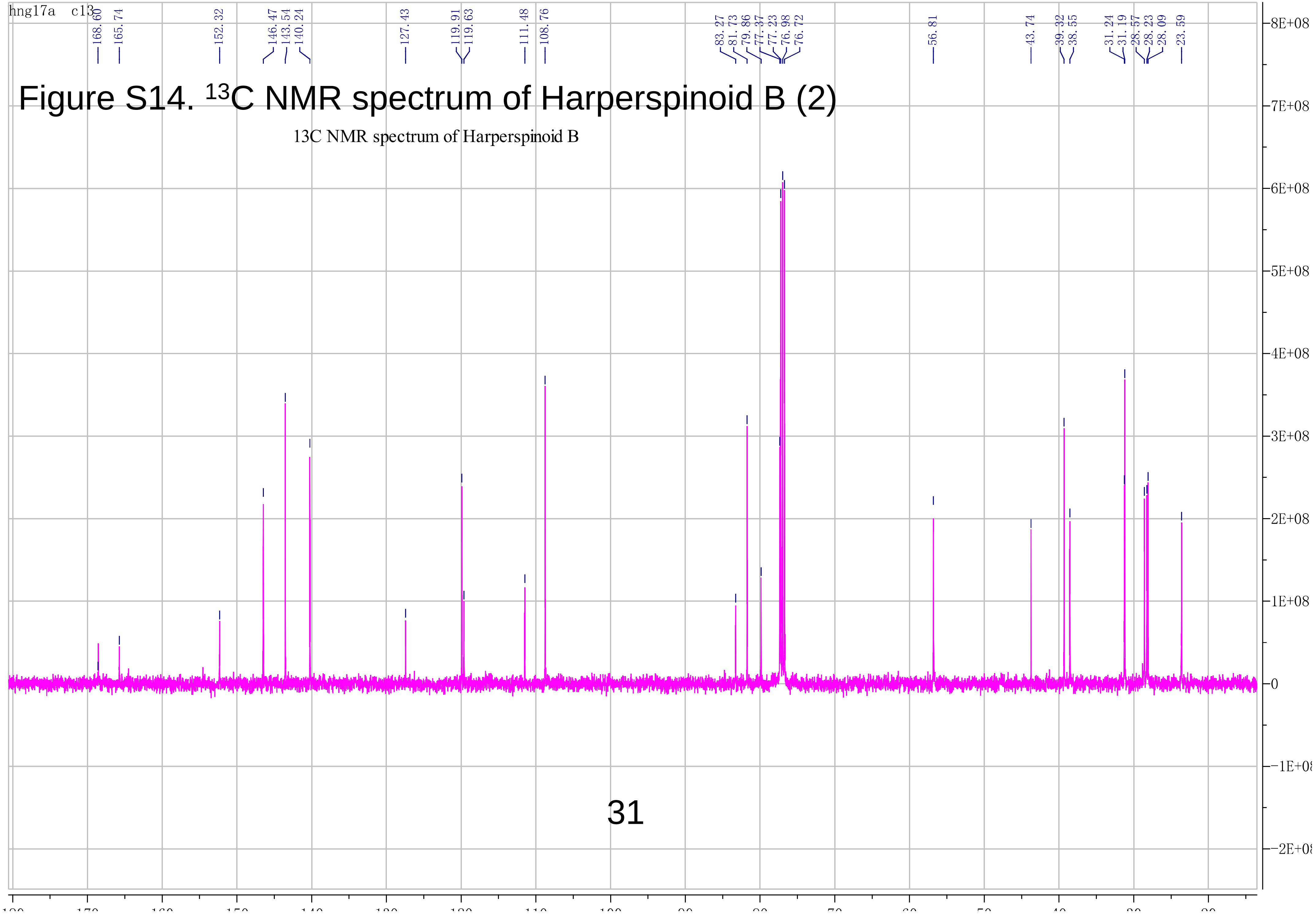

Figure S15. ^{1}H-^{1}H COSY spectrum of of Harperspinoid B (2)

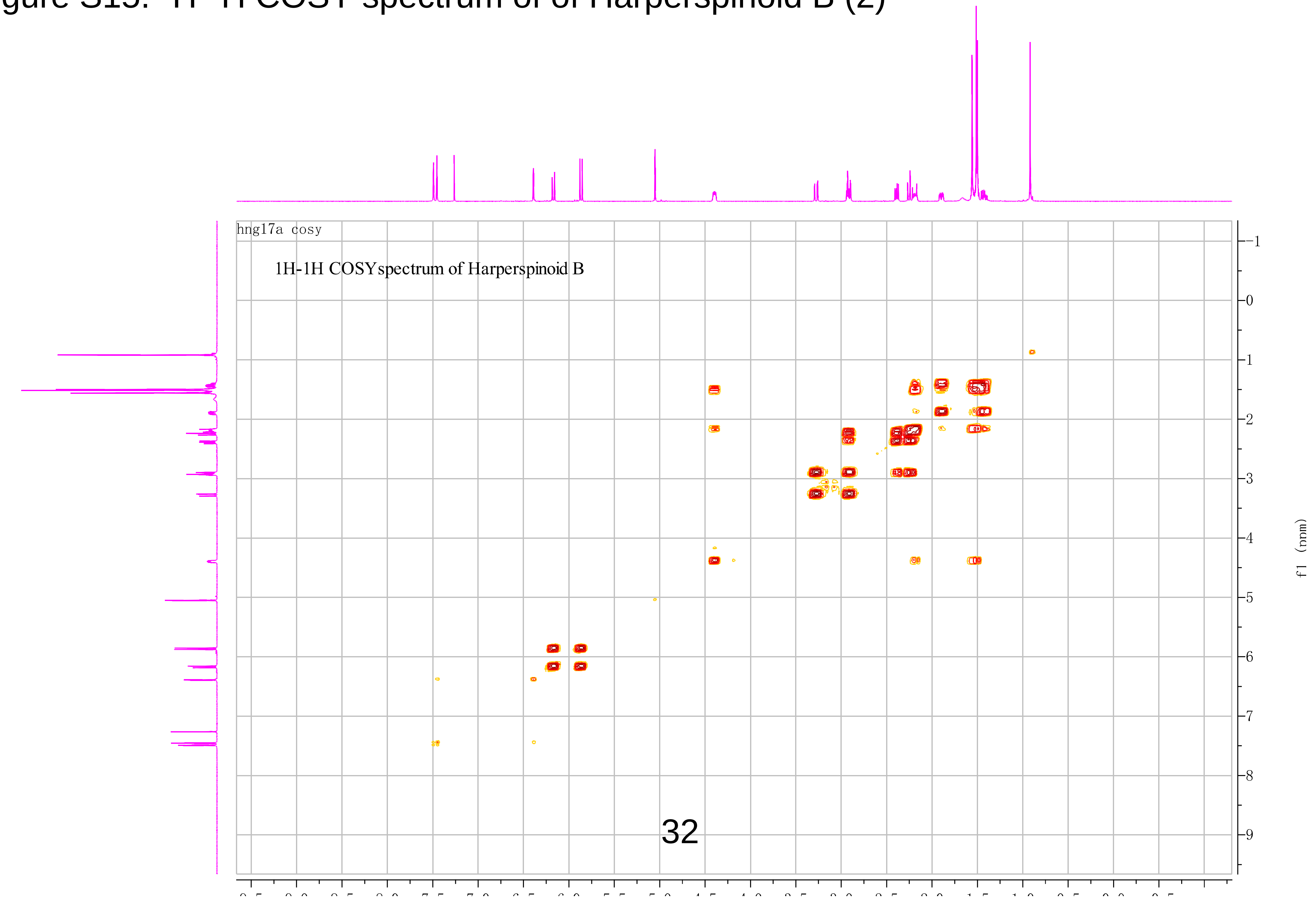

Figure S16. HSQC spectrum of of Harperspinoid B (2)

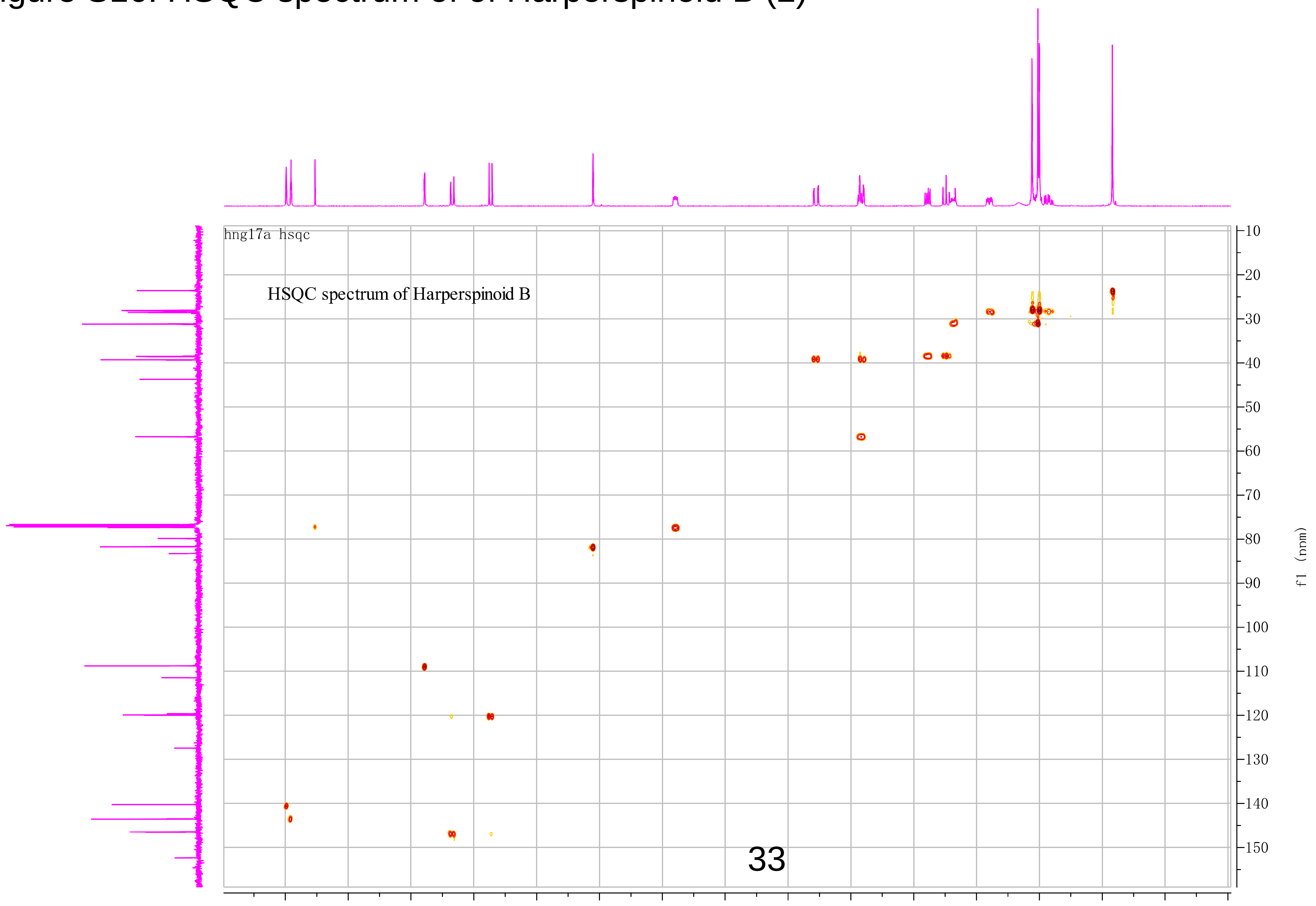

Figure S17. HMBC spectrum of Harperspinoid B (2)

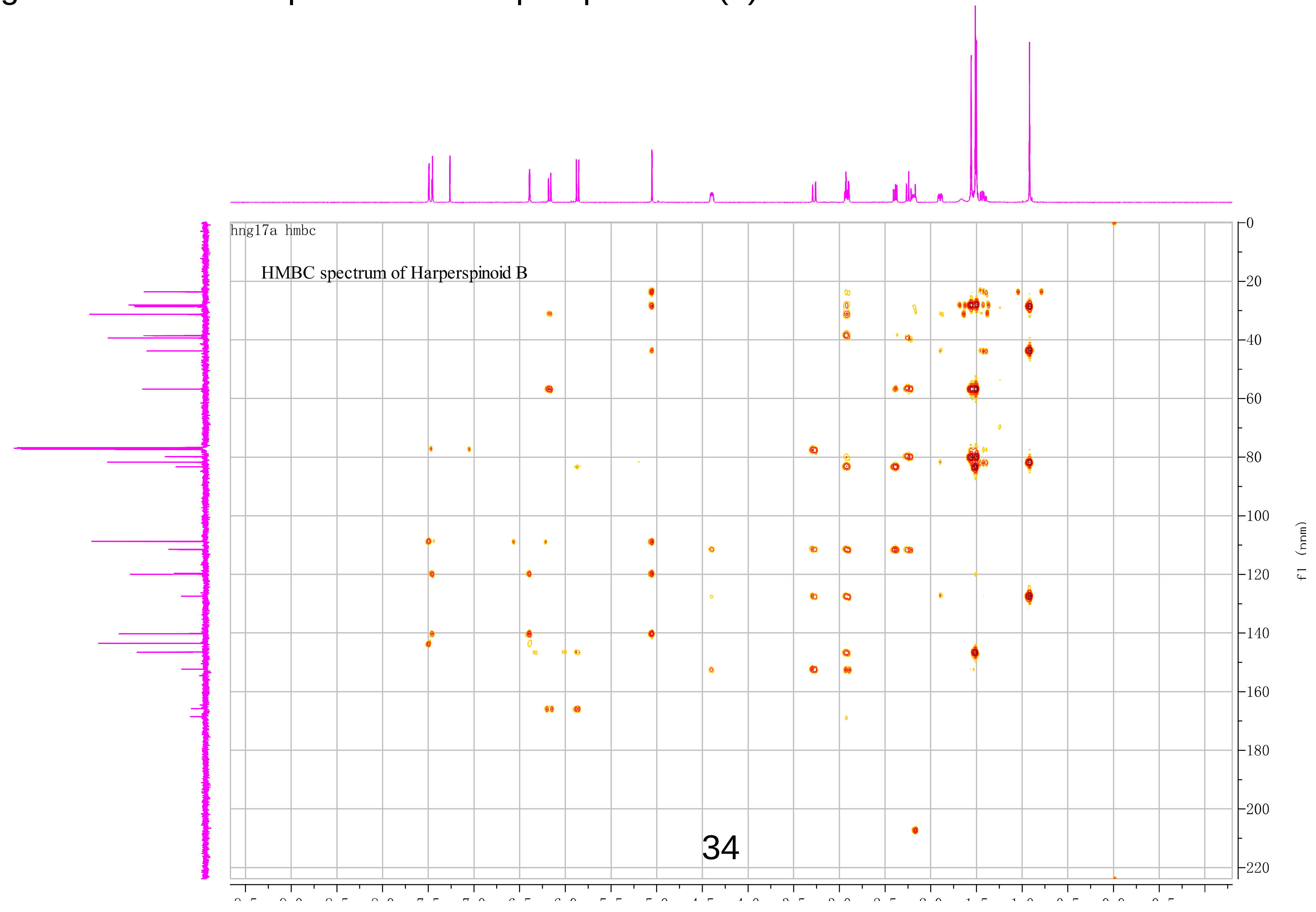

Figure S18. ROESY spectrum of Harperspinoid B (2)

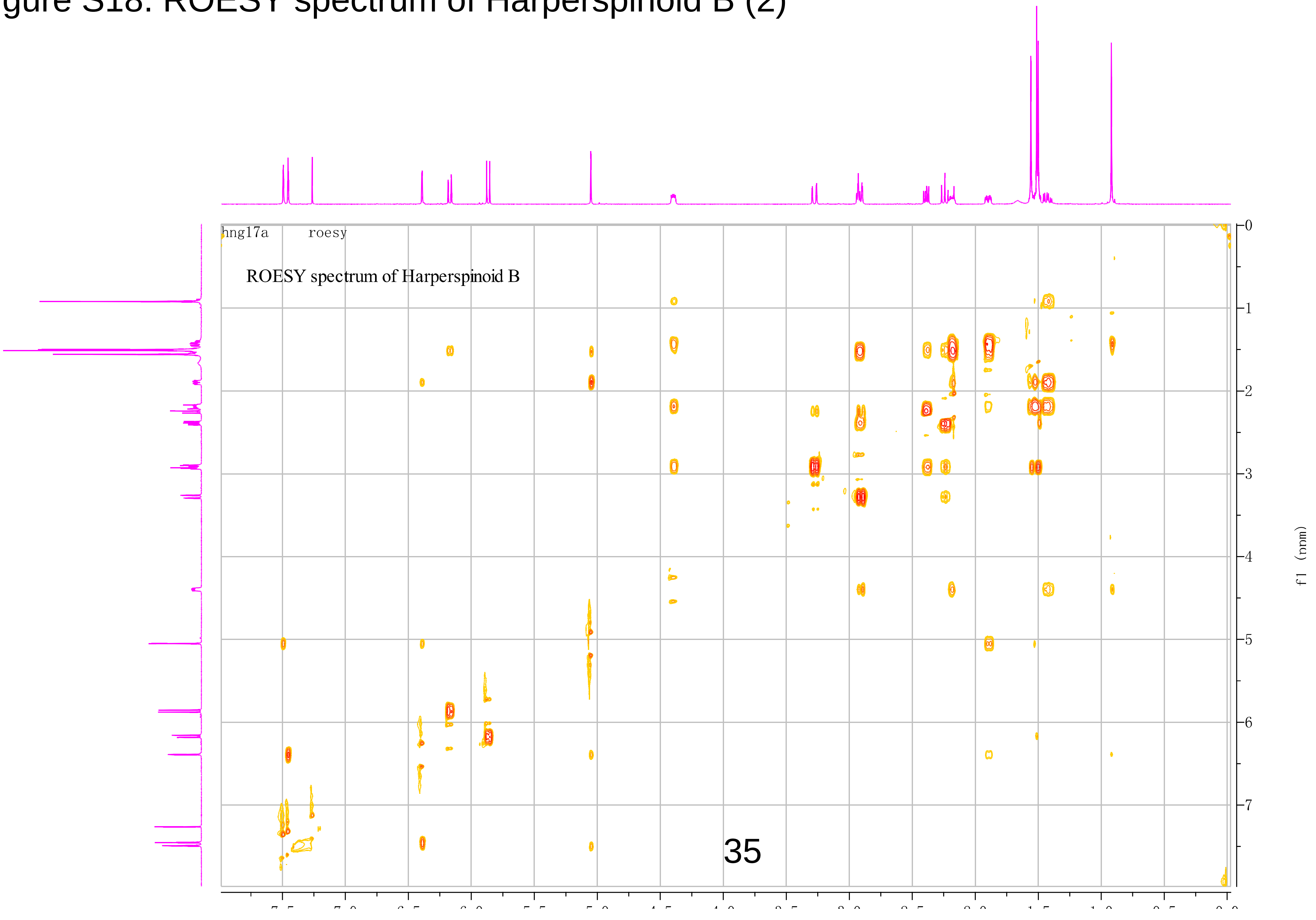

Supplement: Supplementary Information [file srep36927-s1.pdf]
